# Skill-guided agentic reasoning enables longitudinal synthesis of myeloma records at expert-level concordance

**DOI:** 10.1038/s41698-026-01733-2

**Published:** 2026-10-14

**Authors:** Johannes Moll, Jannik Lübberstedt, Christoph Nuernbergk, Jacob Stroh, Luisa Mertens, Anna Purcarea, Christopher Zirn, Zeineb Benchaaben, Fabian Drexel, Hartmut Häntze, Anirudh Narayanan, Friedrich Puttkammer, Andrei Zhukov, Jacqueline Lammert, Sebastian Ziegelmayer, Markus Graf, Marion Högner, Marcus Makowski, Florian Bassermann, Lisa C. Adams, Jiazhen Pan, Daniel Rueckert, Krischan Braitsch, Keno K. Bressem

**Affiliations:** 1 Chair for AI in Healthcare and Medicine, Technical University of Munich (TUM) and TUM University Hospital, Munich, Germany; 2 Technical University of Munich, School of Medicine and Health, TUM University Hospital, Klinikum rechts der Isar, Department of Diagnostic and Interventional Radiology, Munich, Germany; 3 Technical University of Munich, School of Medicine and Health, TUM University Hospital, German Heart Center, Department of Cardiovascular Radiology and Nuclear Medicine, Munich, Germany; 4 Technical University of Munich, School of Medicine and Health, TUM University Hospital, Klinikum rechts der Isar, Department of Medicine III, Munich, Germany; 5 Department of Radiology, Charité – Universitätsmedizin Berlin, corporate member of Freie Universität Berlin and Humboldt Universität zu Berlin, Berlin, Germany; 6 Department of Gastroenterology, Infectious Diseases and Rheumatology, Charité – Universitätsmedizin Berlin, corporate member of Freie Universität Berlin and Humboldt Universität zu Berlin, Berlin, Germany; 7 Chair of Medical Informatics, Institute of Artificial Intelligence in Medicine and Healthcare, TUM School of Medicine and Health, Technical University of Munich, Munich, Germany; 8 Clinical Department of Gynecology, TUM University Hospital, TUM School of Medicine and Health, Munich, Germany; 9 TranslaTUM, Center for Translational Cancer Research, Technical University of Munich, Munich, Germany; 10 Deutsches Konsortium für Translationale Krebsforschung, Heidelberg, Germany; 11 Bavarian Cancer Research Center, Munich, Germany; 12 Department of Engineering Science, University of Oxford, Oxford, UK; 13 Munich Center for Machine Learning (MCML), Munich, Germany; 14 Department of Computing, Imperial College London, London, UK; 15 National Center for Tumor Diseases West, Essen, Germany; 16 Institute of Artificial Intelligence in Medicine, University Hospital Essen, Essen, Germany; 17 Department of Diagnostic and Interventional Radiology and Neuroradiology, University Hospital Essen, Essen, Germany

**Keywords:** Health care, Medical research, Oncology

## Abstract

Patients with multiple myeloma accumulate hundreds of clinical documents over years of sequential therapy, and every treatment decision depends on synthesis across that history. Whether large language model–based systems can match expert agreement on this task, and where they fail, has not been established. On longitudinal records of 811 patients treated over 25 years (44,962 documents and 1,334,677 laboratory values), an agentic reasoning system guided by an indexed clinical skill library was compared against single-pass retrieval-augmented generation (RAG), iterative RAG, iterative RAG augmented with the same skill library, and full-context input on 469 expert-adjudicated patient-question pairs, with an exploratory cross-dataset evaluation on MIMIC-IV. The skill-guided agent exceeded the ceiling at which iterative RAG (75.7%) and full-context input (75.8%) converged, reaching 80.1% concordance, with its advantage widening on the most complex tasks (+11.2 percentage points) and longest records. Providing the same skill library to iterative RAG matched the agent in aggregate (79.0%) and on shorter records, whereas the agent retained a significant advantage on the longest records (+10.3 percentage points in the top decile, *p* = 0.017) and a numerical, non-significant advantage on the most complex questions (+5.4 percentage points). The aggregate error rate (12.2%) was comparable to expert disagreement (13.6%), but a higher proportion of system errors were clinically significant (57.8 versus 18.8%).

## Introduction

Multiple myeloma is managed through sequential lines of therapy over years to decades. Every treatment decision depends on a cumulative record of prior exposures, documented responses, and evolving comorbidities that no single document can fully reflect^1^. The correct interpretation of any individual finding turns on temporal relationships that may span years^2^, and synthesis across document types and timepoints is required at every clinical decision point. The cognitive load of this synthesis falls on hematologists, who already carry among the highest electronic health record burdens of any specialty^3^, and it scales with exactly the patients in whom decision-making matters most, those with the longest and most complex trajectories.

Large language models (LLMs) have been proposed as a way to absorb part of this burden^4^. Published approaches fall into three broad strategies: single-pass retrieval-augmented generation (RAG), iterative RAG, and agentic systems that reason over specialized tools^5–7^. Each has been validated on tasks where the answer can be located in a small number of documents or rows, including guideline interpretation, factual verification, and structured-database question answering^8–11^. Whether the same approaches generalize to the harder regime, in which the answer must be assembled from years of partially contradictory unstructured documentation, has remained an open question. Myers and colleagues recently sharpened it by showing that RAG and full-context input converge to indistinguishable performance at 128,000 tokens on hospitalized-patient tasks, and by attributing the absence of any harder test to the lack of expert-adjudicated longitudinal datasets^12^.

Agentic approaches have been evaluated primarily on diagnostic dialog with simulated patients^13,14^ and on tool use against structured databases where each task is resolvable from a small number of entries^15–17^. Neither setting captures the demand of answering a treatment planning question from multiple instances of unstructured, partially contradictory longitudinal documentation. No evaluation of any such approach has been reported for real multiple myeloma patients or any comparable disease trajectory, and whether clinically reliable performance is achievable against an independently annotated reference standard has not been established.

Here, this gap is addressed by evaluating longitudinal records from 811 patients with multiple myeloma treated at the Technical University of Munich (TUM) University Hospital over 25 years, spanning 44,962 clinical documents and 1,334,677 laboratory values. Clinical questions were stratified into three pre-specified complexity levels reflecting the synthesis demands described above: single-record lookup (Level 1), temporal reasoning across multiple documents or timepoints (Level 2), and criteria-based synthesis against a clinical decision rule, such as therapy eligibility (Level 3). Against this benchmark, an agentic reasoning system guided by an indexed clinical skill library was compared with single-pass RAG, iterative RAG, iterative RAG augmented with the same skill library, and full-context input, all using the same locally deployed open-weight model. Every answer was scored against an independently annotated, expert-adjudicated reference standard, residual inter-rater variability was explicitly classified, and the clinical safety profile was characterized through structured error severity analysis. Transferability was explored in a cross-dataset evaluation on the MIMIC-IV critical care database^18,19^, and all data remained within institutional infrastructure throughout.

## Results

### Skill-guided agents exceed a non-agentic performance ceiling

**Fig. 1:**
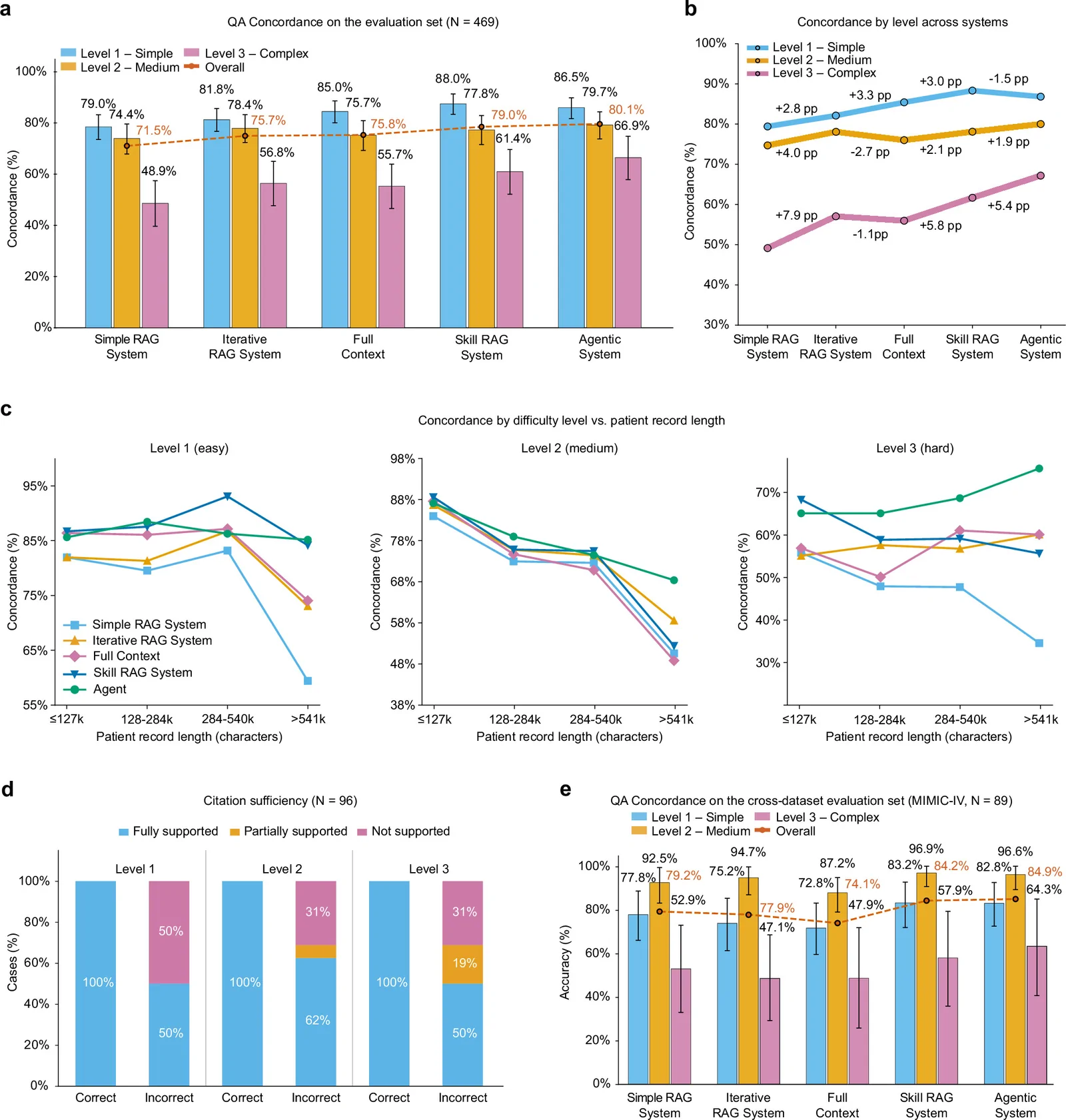
Skill-guided agentic reasoning improves accuracy, with the largest gains in clinically complex tasks and long records requiring longitudinal synthesis. **a** Overall and stratified concordance with expert consensus across system configurations on the primary evaluation cohort, including Iterative RAG augmented with the agent’s clinical skill library (Skill RAG). **b** Concordance by complexity level across systems, annotated with the difference between adjacent systems in percentage points (no adjacent difference reached Bonferroni-corrected significance; pairwise tests are reported in Supplementary Table 3). **c** Concordance stratified by patient record length and complexity level, showing convergence of all systems for shorter records and the largest agentic advantage among patients in the top decile of record length (>541k characters; exploratory, *n* = 10 patients), where all non-agentic configurations, including Skill RAG, decline sharply. **d** Citation sufficiency analysis showing the proportion of responses fully, partially, or not supported by retrieved source documents across complexity levels and concordance status. **e** Exploratory cross-dataset evaluation on the MIMIC-IV cohort (*n* = 20 patients, 89 pairs). The ranking of approaches was numerically preserved, with overlapping confidence intervals.

The primary evaluation comprised 469 expert-adjudicated patient-question pairs across 100 sampled patients, including 200 local lookup questions (Level 1), 179 temporal synthesis questions (Level 2), and 90 criteria-based longitudinal synthesis questions (Level 3). Among the non-agentic approaches, Iterative RAG and Full Context converged at nearly identical performance, reaching 75.7% (95% CI [72.4–79.0]) and 75.8% ([72.6–79.1]) concordance with expert consensus, respectively (−0.1 percentage points, Bonferroni-corrected *p* = 1.00). Simple RAG trailed at 71.5% ([67.8–75.3]). The skill-guided agent exceeded all three approaches, reaching 80.1% ([77.0–83.1]) concordance (Fig. 1a and Supplementary Table 3). This corresponded to a gain of +4.3 percentage points over Full Context ([1.9–6.8], *p* = 0.004) and +4.4 percentage points over Iterative RAG ([1.7–7.3], *p* = 0.014). Performance was stable across ten independent runs, with a standard deviation of 1.4 percentage points (Supplementary Table 4).

This advantage could reflect the agent’s clinical skill library, a set of question-type-specific reasoning protocols selected at runtime, rather than agentic execution itself. Iterative RAG was therefore augmented with the same runtime-selected skill modules, without tool-use planning, iterative tool execution, structured memory state, or deterministic scoring tools. This skill-augmented Iterative RAG reached 79.0% ([75.5–82.3]), 3.3 percentage points above Iterative RAG ([1.5–5.3], *p* = 0.002) and not significantly different from the agent (−1.1 percentage points, [−3.6 to +1.2], *p* = 1.00). Conversely, removing the skill library from the agent reduced concordance by 3.0 percentage points to 76.6%, whereas removing type and date filters in retrieval tools, deterministic clinical scoring tools, the structured memory state, or pre-planned tool use each changed concordance by at most 0.4 percentage points (Supplementary Table 5). Loading all 42 skill modules unconditionally instead of selecting them at runtime changed concordance by less than one percentage point on either architecture (Supplementary Table 5). In aggregate, the skill library therefore accounted for the gain over the non-agentic ceiling regardless of the architecture that executed it. The two architectures separated only when the results were stratified by question complexity and record length, as described below.

In an exploratory cross-dataset evaluation, the systems were applied without adaptation to 89 patient-question pairs across 20 patients from MIMIC-IV (Fig. 1e). The skill-guided agent achieved 84.9% concordance ([77.8–91.2]), compared with 79.2% ([71.4–86.3]) for Simple RAG, 77.9% ([70.1–85.3]) for Iterative RAG, and 74.1% ([65.5–82.2]) for full context. Skill-augmented Iterative RAG reached 84.2% ([78.4–89.2]), matching the agent, with the same direction of difference at Level 3 as in the primary cohort (57.9 versus 64.3%, not significant). The ranking of approaches was therefore numerically preserved despite changes in documentation language, institutional conventions, record structure, and de-identification procedures. With 20 patients, however, the confidence intervals of all approaches overlapped, and this analysis should be read as hypothesis-generating rather than as validation.

### Agentic advantage concentrates on longitudinal synthesis tasks

The central result was not a uniform gain across all questions, but a separation that emerged as the task shifted from retrieval to synthesis. Concordance decreased with question complexity across all approaches (Fig. 1a). For the skill-guided agent, concordance was 86.5% ([82.0–90.7]) for local lookup, 79.7% ([74.4–84.8]) for temporal synthesis, and 66.9% ([58.2–75.1]) for criteria-based longitudinal synthesis. The advantage over Full Context increased monotonically across these levels, from +1.5 percentage points at Level 1 (*p* = 1.00), to +4.0 percentage points at Level 2 (*p* = 0.24), and +11.2 percentage points at Level 3 ([4.8–18.1], *p* = 0.007) (Fig. 1b). A similar gradient was observed relative to Iterative RAG, with no statistically significant difference at Levels 1 or 2 but a +10.1 percentage point advantage at Level 3 ([3.3–17.1], *p* = 0.026). At Level 3, the non-agentic approaches ranged from 48.9 to 56.8% concordance, skill-augmented Iterative RAG reached 61.4%, and the skill-guided agent 66.9%.

Record length produced the same conditional pattern. In a hypothesis-driven exploratory analysis, patients were stratified by total clinical record length into three bins spanning the lower 90th percentile and a top decile above 541k characters (*n* = 10 patients; Supplementary Table 9). In the lower three bins, the skill-guided agent did not differ statistically from Iterative RAG. In the top decile, it achieved 76.9% concordance, compared with 65.1% for Iterative RAG (+11.8 percentage points, [5.1–18.7]) and 61.8% for Full Context (+15.1 percentage points, [9.4–21.7]) (Fig. 1c). This stratification was pre-specified as exploratory and the top decile comprises ten patients, so these differences require confirmation. The degradation pattern was nevertheless consistent with the complexity analysis. From the shortest to the longest record-length bin, concordance fell by 5.5 percentage points for the skill-guided agent, compared with 13.8 points for Iterative RAG, 17.4 points for skill-augmented Iterative RAG, 19.6 points for Full Context, and 26.5 points for Simple RAG.

The comparison with skill-augmented Iterative RAG located the agentic contribution within this pattern (Supplementary Table 3). At Level 1, skill-augmented Iterative RAG was numerically higher than the agent (88.0 versus 86.5%), at Level 2 the difference reversed (−1.9 percentage points), and at Level 3 the agent was 5.4 percentage points higher ([−12.0 to +1.1], *p* = 0.66), so that Level 3 concordance rose in two comparable, individually non-significant steps from Iterative RAG (56.8%) through skill-augmented Iterative RAG (61.4%) to the agent (66.9%). Stratified by record length, the skill library accounted for the gain over Iterative RAG on the shortest records (+4.6 percentage points, [1.3–8.0], *p* = 0.046) but not on the longest (+1.5 percentage points, *p* = 1.00), whereas the agent did not differ from skill-augmented Iterative RAG on the shortest records (−1.6 percentage points, *p* = 1.00) and exceeded it on the longest (+10.3 percentage points, [4.2–17.5], *p* = 0.017). Iterative RAG with all 42 skill modules loaded unconditionally showed the same pattern (68.4 versus 76.9% in the top decile).

The advantage also depended on the backbone model (Supplementary Table 6). Across five locally deployable models, the agent had the highest concordance for three. For gemma-4-31B-it, iterative RAG was higher (82.4 versus 81.6%), and for Qwen3-Next-80 B-A3B-Instruct, the difference was 0.3 percentage points.

### Clinical error profile and failure mechanisms

We next examined whether higher concordance translated into a clinically acceptable error profile. Error classification was performed on a single evaluation run with 77.8% overall agent concordance, below the ten-run mean of 80.1%, providing a conservative basis for safety analysis. All 115 patient-question pairs in which the agent diverged from the expert reference were classified by a senior hematologist into six categories (Table 1). Thirty-three divergences (28.7%) were clinically significant errors, and 24 (20.9%) were clinically insignificant errors. The remaining divergences were partially correct, acceptable or ambiguous, annotation errors, or pipeline failures. The clinically significant error rate was therefore 33 of 469 evaluable pairs (7.0%). Blinded re-annotation by an independent rater yielded a six-category *κ* of 0.667 (95% CI [0.510–0.833]) and a three-stratum *κ* of 0.802 ([0.687–0.962]), indicating substantial to near-perfect reproducibility of the severity classification.

**Table 1:** Error classification of agentic system divergences from expert consensus

Classification	Overall	Level 1	Level 2	Level 3
Clinically significant error	33 (28.7%)	14	12	7
Clinically insignificant error	24 (20.9%)	5	11	8
Partially correct	6 (5.2%)	1	5	0
Acceptable / ambiguous	39 (33.9%)	9	12	18
Annotation error	11 (9.6%)	3	6	2
Pipeline failure	2 (1.7%)	1	0	1
Total	115	33	46	36

All 115 patient-question pairs for which the agentic system output diverged from the adjudicated reference annotation on a single evaluation run (overall concordance 77.8%) were classified by a senior hematologist. The clinically significant error rate was 33 of 469 evaluable pairs (7.0%).

**Fig. 2:**
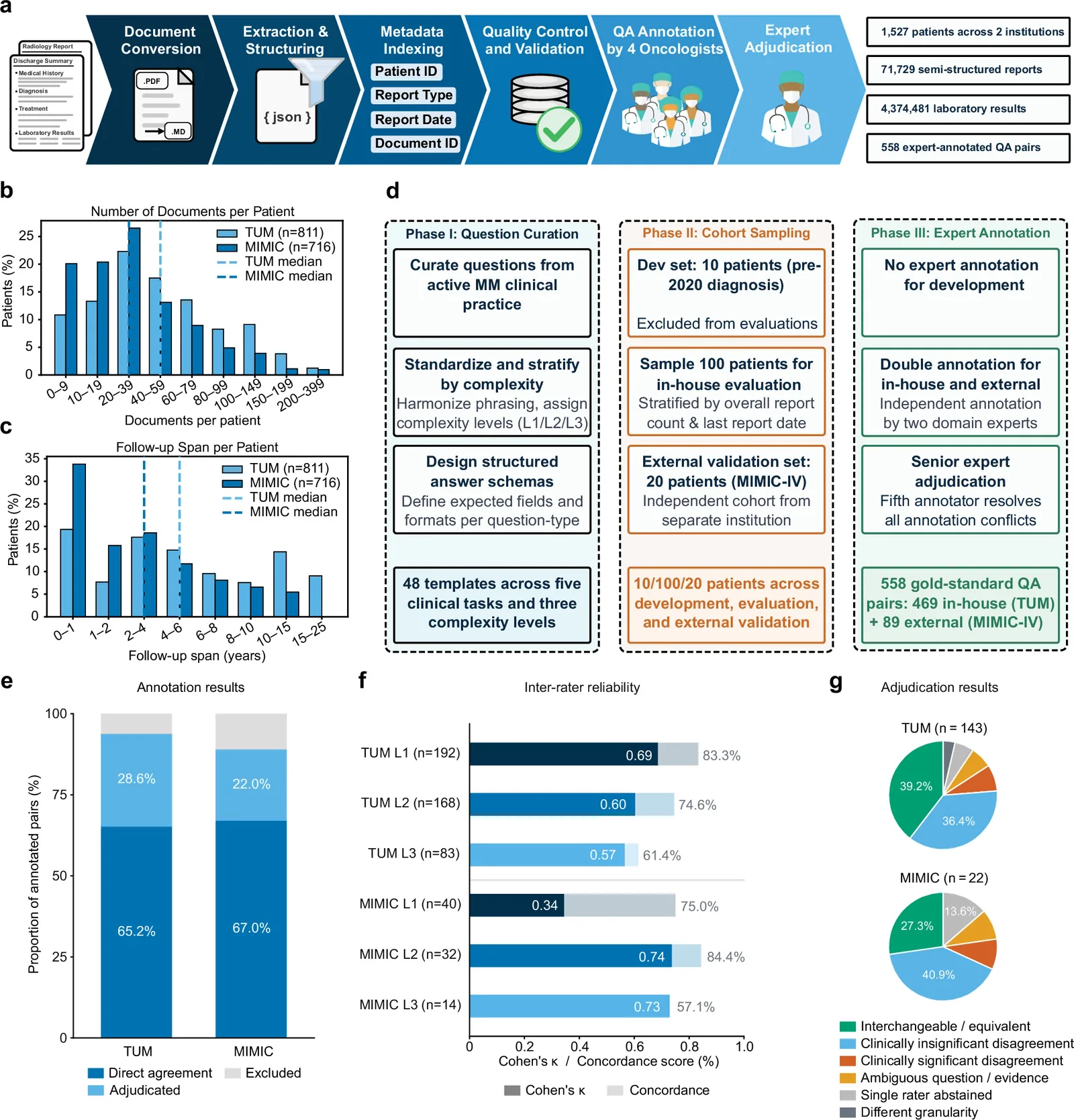
Construction of longitudinal cohorts and an expert-annotated evaluation dataset enabling clinically grounded assessment of longitudinal reasoning. **a** Overview of data sources and preprocessing pipeline across two institutions, including document extraction, structuring, metadata indexing, and quality control applied to heterogeneous clinical records. **b** Distribution of document counts per patient, demonstrating substantial variability in record density and reflecting the complexity of real-world longitudinal documentation. **c** Distribution of follow-up duration, highlighting long-term disease trajectories in the TUM cohort compared with shorter observation windows in MIMIC-IV. **d** Study design and cohort construction, including development, in-house evaluation, and cross-dataset evaluation sets. **e** Annotation outcomes showing proportions of direct agreement, adjudicated cases, and exclusions. **f** Inter-rater reliability across predefined complexity levels, reported as Cohen’s *κ* and observed agreement, illustrating moderate agreement for clinically complex tasks. The low *κ* at MIMIC Level 1 reflects a high prevalence of negative responses, inflating the chance-agreement baseline. **g** Distribution of adjudication categories, indicating that a substantial proportion of disagreements reflects clinically insignificant or interchangeable interpretations rather than true errors.

The total system error rate, combining clinically significant and clinically insignificant errors, was 57 of 469 pairs (12.2%). This was close to the rate of expert disagreement on the same tasks before adjudication (64 of 469 pairs, 13.6%; Fig. 2g). However, the severity profile differed. Among system errors, 57.8% (33 of 57) were clinically significant, compared with 18.8% (12 of 64) among expert disagreements. Thus, the aggregate frequency of errors approached the range of human disagreement, but residual system errors carried greater clinical consequences.

Citation sufficiency analysis separated answer correctness from evidence support. A stratified sample of 96 agent responses from the same run was reviewed, balanced across complexity levels and concordance status, with each of the 48 question templates represented at least once (Fig. 1d). All concordant responses were rated as fully supported by the retrieved source documents. Among discordant responses, full support was present in 50% at Level 1, 62% at Level 2, and 50% at Level 3. Of the 22 responses with insufficient citation support, incomplete retrieval was the dominant failure mechanism, accounting for 83% of fully unsupported responses (*n* = 18). In the remaining 17%, a relevant document had been retrieved but was not incorporated into the final answer. The residual failure modes are therefore separated into two engineering targets: retrieval coverage for missing evidence and synthesis reliability when relevant evidence had already been retrieved.

## Discussion

A clinical AI system answered treatment-relevant questions on years of real myeloma documentation at concordance with expert oncologists, with an aggregate error rate within the range of disagreement between the oncologists themselves. Retrieval-augmented generation and full-context input, two strategies that have dominated published work on language models over clinical records, converged on a shared performance ceiling. Supplying either architecture with encoded clinical reasoning protocols lifted it above that ceiling, but only agentic execution of those protocols retained its advantage on the longest disease trajectories, the cases in which accurate synthesis carries the greatest clinical weight and chart review consumes the most physician time.

The shared performance ceiling that retrieval-augmented and full-context approaches reached indicates that the limiting factor on longitudinal synthesis is not the quantity of evidence presented to the model. Two further components each contributed, and they were separated by record length. Encoded clinical reasoning protocols, delivered as prompt content to iterative RAG, accounted for the aggregate gain and the entire gain on the shortest records, where the relevant evidence is sparse enough for a single generation step to integrate once the decision rule is made explicit. On the longest records the same protocols added nothing, and only their execution through explicit planning, iterative evidence gathering with re-query on insufficient results, structured memory, and deterministic scoring calculators recovered concordance (+10.3 percentage points over skill-augmented iterative RAG in the top decile). RAG and full-context approaches delegate decomposition, evidence weighting, and clinical decision rules to a single generation step, and the present results indicate that this becomes limiting only when the volume of documentation to be integrated exceeds what that step can reliably attend to. This architectural choice is supported by evidence that LLMs are unreliable for clinical calculations unless paired with task-specific tools^20^ and by hybrid neuro-symbolic systems that combine LLMs with rule-based reasoning for auditable clinical information extraction^21^. Within the agent, the skill library is the largest individually separable component (3.0 percentage points when removed), and the remaining components contribute at most 0.4 percentage points in isolation, but the comparison with skill-augmented iterative RAG shows that their joint effect on long records is not captured by removing them one at a time. The convergence previously observed on simpler clinical question answering^12^, therefore, extends to the harder longitudinal synthesis regime, and the present results identify encoded clinical protocols as the intervention required to break it in aggregate and their structured execution as the intervention required on the longest records.

The clinical profile of the agentic advantage identifies a coherent deployment target. The gap over the strongest non-agentic approach widened with both question complexity and total record length, concentrating on patients with the most treatment lines, relapse-remission cycles, and accumulating eligibility constraints, and it persisted against iterative RAG supplied with the same clinical protocols only on those patients. A decade-long myeloma trajectory with multiple sequential regimens and evolving comorbidities is simultaneously the case where manual chart review consumes the most physician time and the case where accurate longitudinal synthesis is most decision-relevant.

The system error rate (12.2%) was comparable to the rate of expert disagreement on the same tasks (13.6%), but the severity distributions were inverted. Among system errors, 57.8% were clinically significant, against 18.8% of expert disagreements. The system, therefore, operated within the bounds of human annotator variability in aggregate, while its errors carried greater average clinical consequence. This distinction is not captured by concordance metrics alone and defines the gap between matching expert agreement in aggregate and readiness for clinical deployment^22^. One measurement asymmetry should be noted: expert disagreements were measured before adjudication, whereas system divergences were measured against the finalized reference standard.

The dominant failure mechanism differed with task complexity. Errors on single-record lookup tasks arose predominantly from incomplete retrieval, a pattern directly addressable through retrieval engineering. Errors on multi-criterion synthesis tasks arose when relevant evidence had been retrieved but was not correctly integrated during reasoning, consistent with the documented tendency of long-context language models to underweight information positioned in the middle of their input^23^ and with recent reports that contextual integration, rather than factual recall, dominates clinical LLM failure modes in real-world deployment^24^. The same mechanism likely explains part of the Full Context underperformance, where relevant passages were present in the context window but were not reliably attended to. Both limitations are properties of the underlying language model rather than of the agentic architecture, and stronger long-context models may narrow the gap.

An exploratory cross-dataset evaluation on the MIMIC-IV critical care database numerically preserved both the system ranking and the complexity gradient without adaptation between cohorts. This analysis does not constitute external validation. The MIMIC-IV cohort was identified by ICD code among intensive care admissions and differs from a dedicated hematology cohort in patient selection, documentation density (median 29 versus 50.5 documents per patient), and follow-up (median 3.3 versus 5.5 years). With 20 patients and 89 pairs, the confidence intervals of all approaches overlapped. Validation on larger, prospectively defined hematology cohorts at further institutions remains needed.

This study had several limitations. Most importantly, the evaluation is retrospective, it establishes concordance with an expert reference standard but does not test whether agentic assistance changes clinical decisions or outcomes, which only prospective study with treating clinicians can determine. The comparison was also restricted to locally deployable open-weight models, as institutional data-privacy requirements precluded sending records to proprietary frontier systems. The relative standing of those systems on this task is therefore unknown. A related scope condition emerged within our own comparison, where the agentic advantage was consistent across three of five backbone models but attenuated for the two with the strongest baseline performance (Supplementary Table 6), indicating that the benefit depends on the long-context integration capacity of the underlying model rather than being guaranteed by the architecture alone. The comparison that isolates agentic execution from the skill library is further limited by sample size. At Level 3 (90 pairs) the 5.4 percentage point difference between the agent and skill-augmented iterative RAG has a confidence interval that includes zero, and the significant difference on the longest records rests on the ten patients in the top decile of record length. The direction of both differences was consistent across complexity levels, record-length bins, and the MIMIC-IV cohort, but their magnitude requires confirmation in a larger cohort of patients with long disease trajectories.

The reference standard carries further caveats, though each was constrained in turn. The question bank was developed with hematologists at the primary institution and finalized before system development, so implicit alignment between question design and architecture cannot be excluded. The cross-dataset evaluation on a different institution and clinical domain (MIMIC-IV) partially addresses this. The senior hematologist who drafted the questions also adjudicated annotation disagreements and performed error classification without independent second review and without formal blinding to system performance, which could bias clinical-significance assignments. Blinded re-annotation by an independent rater nonetheless showed substantial to near-perfect reproducibility (*κ* = 0.667 across six categories, 0.802 across three strata). Finally, expert disagreements were classified before adjudication, whereas system divergences were measured against the finalized reference standard, an asymmetry that may modestly favor the system.

Within these bounds, skill-guided agentic reasoning reached a level of concordance with expert consensus on real longitudinal myeloma records that retrieval-augmented and full-context approaches did not, retained its advantage on the longest records when the same clinical protocols were supplied to iterative RAG, and did so with an error rate falling inside the range of inter-expert variability on the same tasks, though its residual errors carried greater average clinical consequence. The practical implication is that the principal barrier to clinical use is no longer aggregate accuracy but the severity profile of the errors that remain, a target that error-aware design and human oversight can address directly. Prospective evaluation in routine care, with clinicians acting on system outputs, will be required to establish whether these results translate into measurable benefit at the point of care.

## Methods

The study was approved by the Ethics Committee of the Technical University of Munich (approval 2024-590-S-CB), and informed consent was waived, given the retrospective design. Use of MIMIC-IV data was conducted under the PhysioNet credentialed data use agreement^25^. All procedures followed the Declaration of Helsinki.

### Study design and data sources

A retrospective evaluation of expert concordance was conducted on longitudinal multiple myeloma records from two independent institutions (Fig. 2a). The primary dataset comprised all patients with a confirmed multiple myeloma diagnosis treated at TUM University Hospital between 1 January 2001 and 1 January 2026 (*n* = 811). Textual reports were extracted from 44,962 German-language clinical documents, yielding a median of 55 documents per patient (IQR 20 to 76, Fig. 2b) and a median follow-up of 6.3 years from diagnosis (IQR 1.7 to 9.7, Fig. 2c). The cross-dataset evaluation set comprised 716 patients identified by ICD code among intensive care admissions in the publicly available de-identified MIMIC-IV critical care database^19^, yielding 26,767 clinical documents. This cohort was used without adaptation in an exploratory analysis of transferability, not as a validation cohort.

#### Document preprocessing and extraction pipeline

Discharge summaries, radiology reports, pathology reports, tumor board proceedings, and ancillary document types (cardiology, cytology, flow cytometry, and genomic diagnostics reports) were exported as PDF files from the institutional SAP electronic health record system at TUM University Hospital and converted to structured Markdown using a rule-based module that applied font, size, positioning, and layout metadata to exclude non-clinical content and to identify section boundaries, which were preserved as hierarchical headers in the output.

A post-processing step merged sections shorter than 50 words into the nearest adjacent section and split sections exceeding 350 words into overlapping chunks with a 50-word overlap. Laboratory reports stored as structured data were handled separately and excluded from text segmentation.

Document-level metadata (patient identifier, report type, report date, and document identifier) were captured at export and stored alongside each section as structured fields, enabling retrieval queries to filter by type, date, or patient without parsing free text. Report type labels were mapped to a controlled vocabulary of nine canonical categories used as filter arguments by the agentic system’s retrieval tool. Sections were indexed using FTS5 full-text search with Porter stemming, and metadata fields were stored as unindexed columns to support type- and date-based filtering without affecting relevance scoring.

#### Laboratory data normalization and concept mapping

Laboratory values were extracted from the institutional SAP system as timestamped observations comprising a test name, a numeric or categorical result, the reporting unit, and the laboratory-defined reference range. Because test names in the source system reflect free-text entry conventions that evolved over more than two decades of clinical use, a two-stage normalization pipeline was developed to enable reliable programmatic retrieval.

In the first stage, a catalog of canonical laboratory concepts was constructed from all test names observed across the myeloma cohort. Each unique institutional code was assigned a stable identifier derived from a deterministic hash of its normalized form. Text normalization comprised case folding, transliteration of German umlauts (*ä* → *ae*, *ü* → *ue*, *ö* → *oe*, *ß* → *ss*), removal of punctuation and special characters, and whitespace collapsing, yielding 731 unique canonical codes.

In the second stage, an alias index was built to resolve synonymous test names to canonical codes. A total of 1054 observed name variants were mapped to 736 canonical target codes, producing 1404 alias entries. Of the canonical codes, 228 (31%) were associated with two or more synonymous input names, reflecting institutional naming changes over time, the coexistence of abbreviated and full-length designations (e.g., *crp (c-reakt. pr)* and *crp (c-reakt. protein)*), and specimen-type suffixes (e.g., *albumin*, *albumin sm*, *albumin l*). A set of search terms was pre-computed per canonical code from tokenized variants to support fuzzy matching at query time.

The resulting catalog spans myeloma-specific markers (serum and urine immunoglobulin free light chains *κ* and *λ*, free light chain ratio, serum protein electrophoresis, immunofixation, immunoglobulins G, A, M, and D, *β*_2_-microglobulin), prognostic parameters (lactate dehydrogenase, C-reactive protein, calcium, albumin, hemoglobin, creatinine), and the broader set of routine hematological, biochemical, and coagulation tests documented over each patient’s care. No unit conversion was applied; the original test name, reporting unit, and institutional reference range were returned alongside each numeric result. Laboratory values were indexed by their original timestamps with no temporal windowing imposed.

### Clinical question bank and evaluation cohorts

**Fig. 3:**
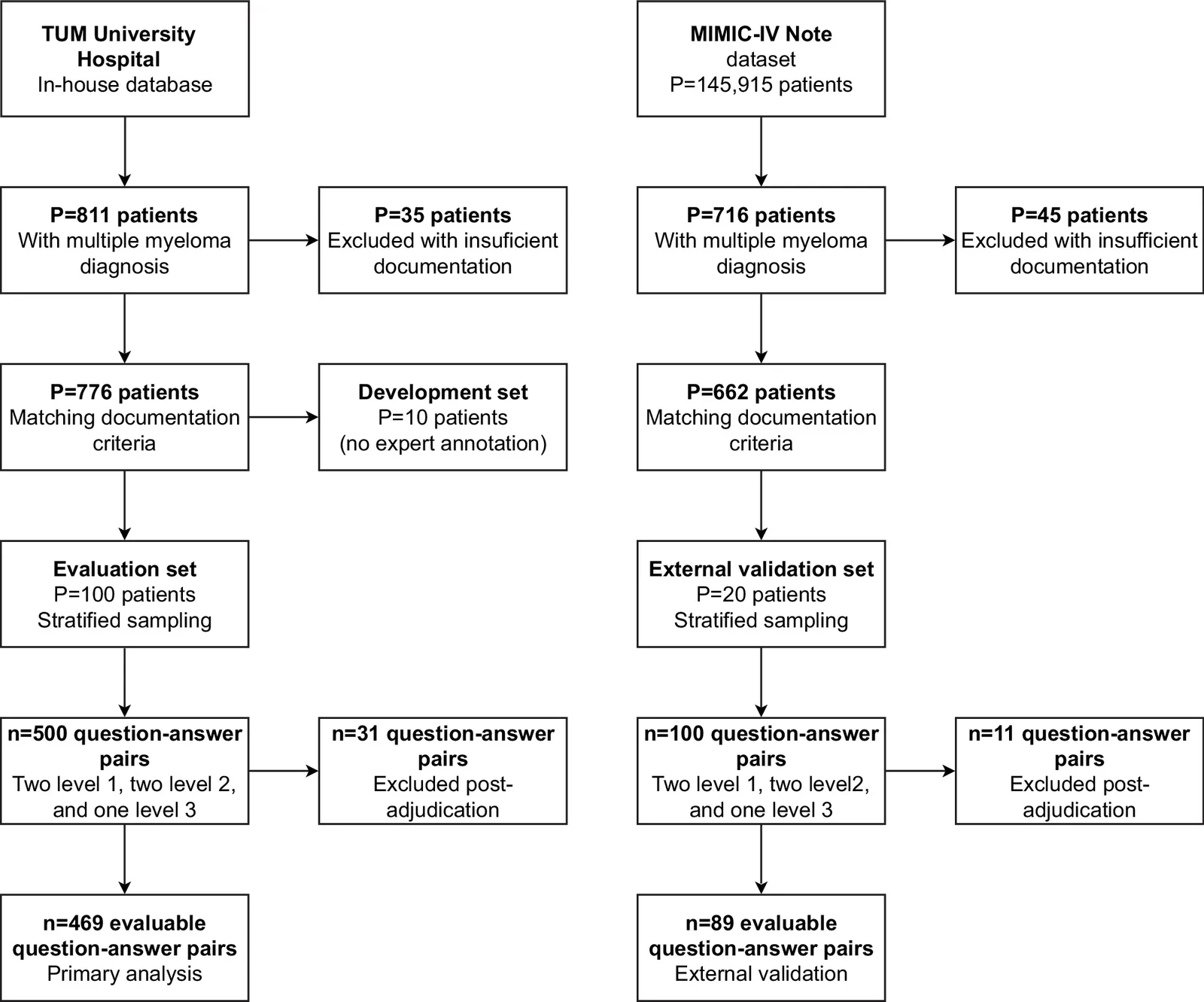
Cohort selection yields representative evaluation sets for longitudinal clinical reasoning tasks. Flow diagram of patient inclusion from institutional (TUM) and external (MIMIC-IV) datasets, including filtering based on diagnosis and documentation criteria. The primary evaluation cohort comprised 100 patients with 500 annotated question–answer pairs, of which 469 were retained after adjudication. The cross-dataset evaluation cohort (MIMIC-IV) included 20 patients with 100 annotated pairs, of which 89 were retained. Stratified sampling ensured coverage across varying documentation density and temporal extent, supporting evaluation of reasoning under heterogeneous real-world conditions.

Three non-overlapping patient sets were defined prior to system development (Figs. 2d and 3). A development set of ten patients was excluded from all reported evaluations. For the primary analysis, 100 patients were sampled from the remaining TUM cohort using stratified sampling, with each patient assigned five questions (two Level 1, two Level 2, one Level 3), yielding 500 patient-question pairs. For the cross-dataset evaluation, 20 patients were selected from the MIMIC-IV cohort using the same procedure, yielding 100 pairs.

#### Clinical question bank: development and instantiation

The clinical question bank was developed by a senior hematologist (KB) in consultation with the annotating oncologists (AP, CN, JS, and LM) prior to any system development or evaluation. Templates were drafted to cover core clinical decision tasks in the longitudinal management of multiple myeloma, including current and prior treatment status, treatment response assessment, toxicity and dose modification documentation, diagnostic workup completion, disease staging, comorbidity scoring, and therapy eligibility determination. The initial template set was revised through iterative consensus discussion until coverage of the intended task space was judged representative. All revisions were completed before the evaluation cohort was defined and before any system outputs were generated.

Each template was assigned by consensus to one of three complexity levels based on the cognitive operations required to produce a correct answer. Level 1 (single-record lookup) comprised questions answerable from a single document or structured data entry, such as whether the patient was currently receiving a specific medication. Level 2 (temporal reasoning) required integration of information across multiple documents or timepoints, such as identifying all documented treatment intervals for a given agent or determining the best documented response under a specified regimen. Level 3 (criteria-based synthesis) required structured synthesis across document types, laboratory values, and clinical timepoints against a multi-criterion decision rule, such as evaluating eligibility for BCMA-directed CAR-T-cell therapy. The final bank comprised 48 templates (20 at Level 1, 18 at Level 2, and 10 at Level 3) spanning five clinical task categories (single choice, treatment intervals, first occurrence, staging, and eligibility). The full template list is provided in Supplementary Table 2.

The answer schema of each template determined the scoring method: binary scoring for single categorical responses (a yes/no/not documented answer, a single date, or a single staging score) and list-type F1 scoring for enumerations (cycle start dates with doses, treatment intervals), as defined under Scoring and concordance computation.

For each patient in the evaluation cohort, five questions were instantiated from the template bank (two at Level 1, two at Level 2, and one at Level 3) by random draw without replacement from a shuffled deck at each complexity level (seed = 42). When the deck was exhausted, it was reshuffled, ensuring balanced reuse across patients. Date placeholders were replaced with the date of the last available clinical report for that patient. For the MIMIC-IV cohort, de-identified dates were used as recorded in the dataset, which applies a consistent per-patient shift that preserves temporal ordering and intervals. Whether a given question was answerable from a particular patient’s record was determined only during expert annotation. The same instantiation procedure was applied to both cohorts.

### Expert annotation and adjudication

All pairs were annotated by independent chart review prior to system evaluation, with raters blinded to each other and to all system outputs. Each TUM patient was reviewed by exactly two of four oncologists with multiple myeloma expertise (AP, CN, JS, and LM). Disagreements were reviewed by a senior hematologist (KB) and classified into one of five categories.

#### Annotation protocol

Prior to annotation, all four oncologists (AP, CN, JS, and LM) received structured training from the senior hematologist (KB) on the question bank, the answer schema conventions, the complexity-level definitions, and the criteria for abstention. Each annotator was assigned 50 patients from the TUM evaluation cohort by randomized balanced allocation such that each patient was reviewed by exactly two annotators and no annotator pair was systematically overrepresented (seed = 42). For the MIMIC-IV cohort, 20 patients were distributed between two of the same annotators using the same allocation procedure.

Annotation was performed using a purpose-built Streamlit web application that presented each question with schema-aware input widgets (single-choice dropdowns, multi-interval entry forms, criteria tables with per-criterion status fields, date entry, and free-text fields). Annotators were instructed to use the abstain checkbox when an answer was not clearly supported by available documentation up to the cutoff date, and could optionally select one or more evidence documents and provide free-text comments. All responses were persisted to CSV logs with timestamps.

For the TUM cohort, annotators reviewed patient records using the institutional SAP interface, which they use routinely in clinical practice. Each question specified a cutoff date; annotators were instructed to disregard documentation dated after the cutoff and, for conflicting statements, to prefer the most recent documentation before that date. For the MIMIC-IV cohort, annotators accessed an equivalent data browser providing report viewing and structured laboratory retrieval over the de-identified database. Annotators were blinded to each other’s responses throughout; the application enforced single-user login and stored responses in separate log files. No model-generated content was displayed at any point during annotation.

#### Adjudication rules and disagreement taxonomy

Patient-question pairs for which two independent annotators did not reach direct agreement fell into one of three types: categorical disagreements (both annotators provided a response but on differing substantive content), single-rater abstentions (one annotator provided a response and the other abstained), and dual abstentions (both annotators abstained). Categorical disagreements and single-rater abstentions were referred to the senior hematologist (KB) for adjudication; dual abstentions were excluded from the evaluable set without adjudication.

The adjudicator reviewed each disagreement using the same record interface and cutoff-date convention as the original annotators. Both annotators’ responses were presented side by side, including evidence selections and comments, and the adjudicator provided a final answer using the same schema-aware interface. The adjudicator could adopt either annotator’s response or provide an independent answer. In single-rater abstention cases, the non-abstaining rater’s response was independently verified rather than accepted by default. Pairs for which the adjudicator also abstained were excluded from the evaluable set.

Each adjudicated disagreement was classified into one of five categories. Interchangeable or equivalent responses covered cases in which both answers were clinically synonymous or differed only in phrasing, abbreviation, or formatting. Clinically insignificant disagreement covered cases in which one annotator made an error or omission that would not alter a clinical decision. Clinically significant disagreement covered cases in which the two annotators reached substantively different clinical conclusions with different management implications. Ambiguous question or evidence covered cases in which the record did not contain sufficient or unambiguous information, or the instantiated question admitted more than one defensible interpretation. The difference in response granularity covered cases in which both annotators were correct but reported at different levels of detail. Single-rater abstentions were adjudicated but not assigned a disagreement category. The adjudicated answer served as the reference standard for all concordance analyses. For pairs with direct agreement, the shared answer was used without adjudication.

#### Expert annotation and adjudication outcomes

For the TUM cohort, direct agreement was reached on 326 of 500 pairs (65.2%). A further 143 (28.6%) were included after adjudication and 31 (6.2%) were excluded (29 dual abstentions and 2 adjudicator abstentions), yielding 469 evaluable pairs in the primary analysis (200 Level 1, 179 Level 2, 90 Level 3; Fig. 2e). Pre-adjudication inter-rater agreement was *κ* = 0.69 at Level 1 (observed agreement 83.3%, *n* = 192), *κ* = 0.60 at Level 2 (observed agreement 74.6%, *n* = 168), and *κ* = 0.57 at Level 3 (observed agreement 61.4%, *n* = 83) (Fig. 2f). Among adjudicated cases, the most frequent disagreement categories were interchangeable or equivalent responses (39.2%) and clinically insignificant disagreement (36.4%), with the remainder attributable to clinically significant disagreement (8.4%), ambiguous question or evidence (7.0%), differences in response granularity (3.5%), and single-rater abstentions (5.6%) (Fig. 2g).

For the MIMIC-IV cohort, direct agreement was reached on 67.0% of pairs. A further 22.0% were included after adjudication, and 11.0% were excluded, yielding 89 evaluable pairs. Pre-adjudication inter-rater agreement was *κ* = 0.74 at Level 2 (observed agreement 81.2%, *n* = 32) and *κ* = 0.73 at Level 3 (observed agreement 78.6%, *n* = 14). The lower Level 1 kappa (*κ* = 0.34, observed agreement 75.0%, *n* = 40) reflects the high prevalence of negative responses in the MIMIC-IV records (72.5% of Level 1 answers were “False”), which inflates the chance-agreement baseline and suppresses *κ* even when observed agreement is high.

### Agentic system and comparators

**Fig. 4:**
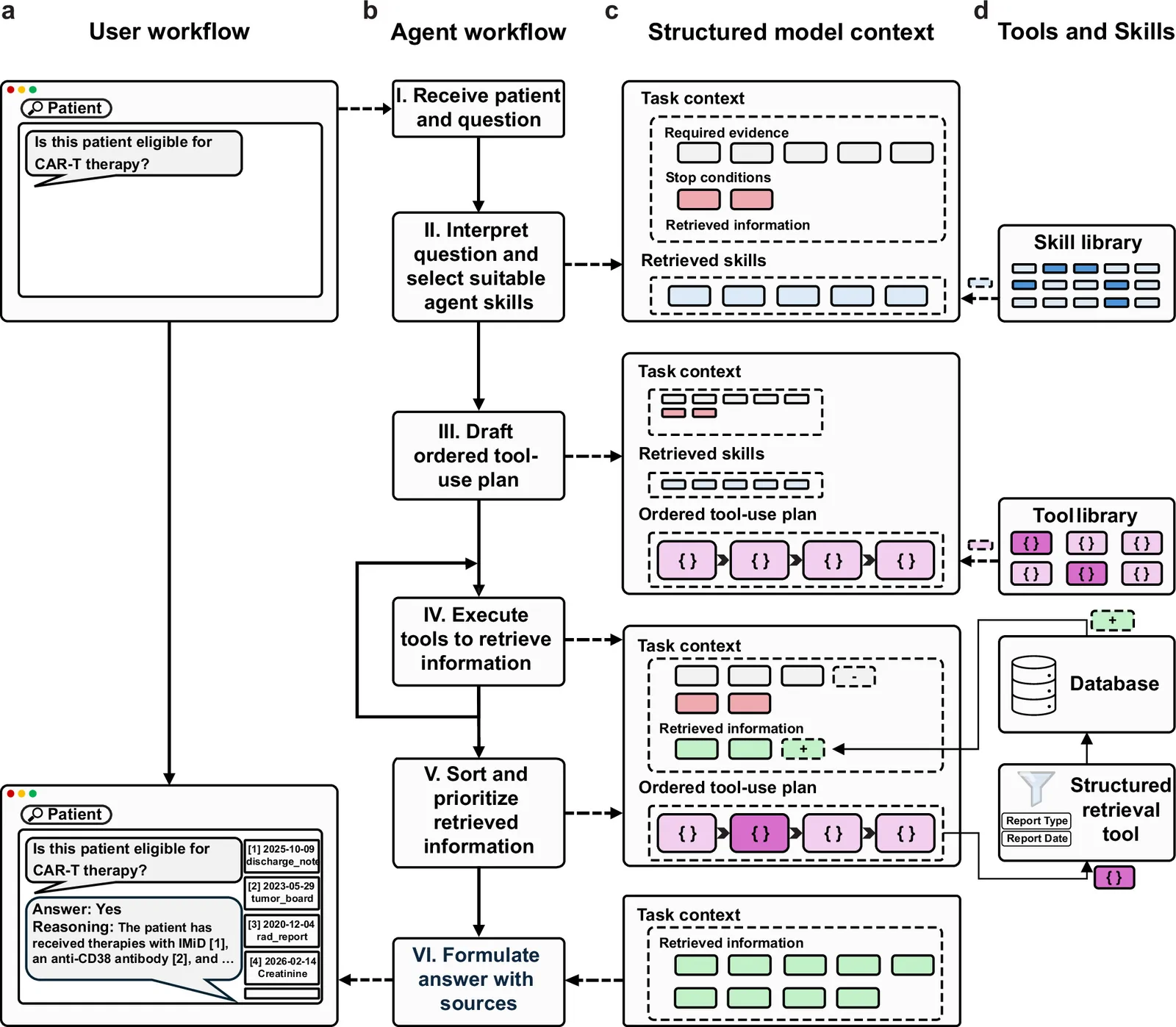
Agentic system enables structured, traceable clinical reasoning across longitudinal patient records. **a** User-facing workflow illustrating query input and generation of citation-backed answers grounded in patient records. **b** Internal agent workflow, including question interpretation, retrieval of task-specific clinical skills, generation of an ordered tool-use plan, iterative evidence retrieval, and synthesis. **c** Structured model context integrating task requirements, retrieved domain knowledge, intermediate evidence, and stopping criteria for reasoning completion. **d** Tool and skill library supporting structured access to clinical reports, laboratory trajectories, and deterministic scoring systems. Together, these components enable multi-step reasoning in which each intermediate step is explicitly linked to retrieved evidence, ensuring that final answers are verifiable and traceable to source documents.

The agentic system was built around an explicit separation of clinical reasoning from language generation, with four architectural components distinguishing it from retrieval-based approaches (Fig. 4). Question-type-specific reasoning protocols were drawn from an indexed clinical skill library and selected at runtime according to the task. An ordered tool-use plan with explicit stopping conditions was constructed prior to evidence retrieval. A structured memory state encoding the query, accumulated evidence, missing information, and stopping conditions was maintained and updated after each step. Tools included report and laboratory value retrieval with type and date filters, alongside deterministic clinical scoring calculators that encoded staging and eligibility rules without delegating them to the language model. Final answers followed a schema-defined format with inline source citations.

The same locally deployed 120-billion-parameter open-weight language model (gpt-oss-120b^26^) was used across all four comparator approaches and the same patient record database. In Simple RAG, single-pass dense retrieval was implemented without query rewriting or reranking^27^. Iterative RAG was extended to include subquery rewriting, hybrid BM25 and dense retrieval fusion, cross-encoder reranking, and a multi-round sufficiency loop^5,28^. Skill-augmented Iterative RAG added the runtime skill selection step of the agent to this pipeline. Before retrieval, the same selection prompt, keyword fallback, and deterministic policy-skill attachment chose modules from the indexed clinical skill library (mean 6.9 modules per question, compared with 6.8 for the agent), and the selected workflow, parsing, and knowledge skills were rendered into the subquery rewriting and answer prompts, style skills into the answer prompt, and policy skills as plain-text instructions into the answer prompt. Workflow skills that reference tool invocations were included as plain text. Retrieval, reranking, the sufficiency loop, the language model, and decoding settings were identical to Iterative RAG, and no tool-use plan, tool execution, structured memory state, policy engine, or scoring calculator was available. In the Full Context configuration, retrieval was bypassed entirely, all documents and laboratory values were concatenated in reverse chronological order until the context window was filled^12^. Two additional comparators are reported in Supplementary Table 3.

#### Agentic system: implementation details

The agentic system was implemented as a four-phase reasoning pipeline. Each phase corresponded to a structured prompt whose output was parsed as JSON and validated before progression to the next phase. The four phases were question assessment and skill selection, tool-use plan construction, iterative tool execution, and final answer synthesis. A schematic overview is provided in Fig. 4.

**Phase I: Question assessment and skill selection**. Upon receipt of a clinical question, the system derived a short medical analysis identifying the clinical intent and key hypotheses, a list of required and currently missing information points, and a preliminary complexity assessment. In parallel, a subset of skill modules was selected from the indexed clinical skill library based on question content and available skill summaries. Heuristic keyword matching served as a fallback to ensure that question-type-specific style skills were activated even when the language model omitted them, for example, questions containing temporal keywords triggered the current-status style skill, and questions referencing eligibility criteria triggered the eligibility style skill. A base structured-annotation style skill was always included to enforce the two-line output format.

**Clinical skill library**. The skill library comprised modular instruction packages in four categories. Workflow skills encoded task-specific decomposition strategies and evidence priorities for clinical tasks such as therapy reconstruction, eligibility assessment, staging score calculation, and laboratory trend interpretation. Parsing skills provided normalization rules for institutional abbreviations, German-language date formats, and entity synonymy (e.g., mapping trade names to generic drug names). Style skills defined answer structure templates for each question type, including required fields and formatting conventions. Policy skills encoded deterministic precedence rules for resolving contradictory evidence, including temporal authority (most recent document takes precedence), plan-versus-administered therapy disambiguation (administered therapy overrides documented plans), and contradiction resolution heuristics.

Skills were indexed by identifier and category. The language model selected relevant skill identifiers from a summary prompt listing all available skills. Selected skills were rendered into two context blocks: a workflow context block (workflow, parsing, and knowledge skills) injected during tool execution, and a style context block (style skills) injected during final answer synthesis. Policy skills were attached deterministically based on the selected style and workflow skills rather than by model selection, ensuring consistent application of temporal authority and contradiction resolution rules regardless of model selection behavior. Across the evaluation cohort, an average of 6.7 skill modules were selected per question, corresponding to ~8000 tokens of skill context, compared with ~49,000 tokens required to load all 42 modules unconditionally. The contribution of the selection mechanism to accuracy and efficiency is reported in the ablation analysis (Supplementary Table 5).

**Fixed baseline prompt**. All system configurations, including ablation conditions in which the skill library was disabled, shared a fixed baseline prompt of approximately 500 tokens. This prompt provided task framing (instructing the model to answer clinical questions from retrieved context only), the two-line output format specification, all answer schema definitions with worked examples covering single-choice, list, multi-interval, cycle-start-dose-list, and criteria-table formats, citation formatting rules using bracketed context identifiers, the distinction between “Not documented” (information absent from the record) and “No” (documented negative finding), and drug name normalization mappings covering drug class to substance mappings (CD38 antibodies, immunomodulatory drugs, proteasome inhibitors, CAR-T, and bispecific T-cell engagers), common abbreviations (Dara, Btz, and Len), and standard regimen names (VRd, KRd, Dara-Rd, and DVRd). Clinical reasoning workflows, temporal reasoning policies, disease-specific reference knowledge (staging criteria and response categories), and structured retrieval strategies were provided exclusively by the skill library, ensuring that ablation comparisons isolated the contribution of clinical reasoning protocols from formatting, schema, and normalization knowledge.

**Phase II: Tool-use plan construction**. A structured tool-use plan was drafted as a JSON object containing an ordered array of steps and a set of global stopping conditions. Each step specified a step number, a natural-language objective, the tool to be invoked, the tool arguments as a JSON object, a list of evidence requirements, and a conditional stopping rule. Up to three plan construction attempts were permitted; if the language model returned an invalid or empty plan, a repair prompt was appended, and the plan was regenerated. If all three attempts failed, the run was terminated as a pipeline failure. In ablation conditions where planning was disabled, tools were selected reactively at each execution step without a pre-drafted plan.

**Phase III: Iterative tool execution**. Across execution rounds, the system maintained a structured memory state as a JSON object encoding the original query, accumulated evidence from prior tool calls, outstanding missing information, and the global stopping conditions defined in the tool-use plan. This state was updated after each round and passed forward to inform tool selection, re-query decisions, and termination.

The plan was executed step by step, with up to eight tool-use rounds permitted per question. At each round, the language model received the current plan step, accumulated evidence from prior rounds, the list of still-missing information, and the set of allowed tools, then selected one of three actions. It could invoke a specified tool, advance to the next plan step without a tool call, or terminate execution and proceed to answer synthesis. If the model chose to terminate before executing at least one tool call for the current step and further planned steps remained, a repair prompt required it to either execute the planned tool or explicitly skip the step.

Duplicate queries (identical tool name and query string) were blocked, and the model was notified. A negative query cache tracked report retrieval queries that returned no results, preventing re-execution of identical unsuccessful queries. Tool results were cached within each run to avoid redundant calls with identical arguments. An automatic adjustment mechanism progressively broadened retrieval parameters after repeated empty results: after one failure, the number of returned results was doubled (up to a cap of 30), after two failures, the query was reduced to its keyword subset, and after three failures, temporal scope restrictions were removed. A context budget of 120,000 tokens was enforced; if the estimated token count of accumulated context nodes exceeded this limit, retrieval was terminated early, and the system proceeded to answer synthesis. Context nodes were deduplicated by section identifier before being passed to the final answer phase.

**Tool specifications**. Five tool classes were available to the agentic system. The report retrieval tool performed a keyword search over the document database using BM25 ranking (BM25Okapi from the rank_bm25 library). The tool accepted a free-text query string, a report type filter selecting from nine canonical document categories, a temporal scope (all records, the most recent document, a specific date, or a date range), and corresponding date parameters. A report type synonym mapping normalized common German-language and abbreviated report type names to canonical values, and query strings were sanitized to remove answer-schema tokens that the language model occasionally included in retrieval queries.

The laboratory query tool fetched structured laboratory values by canonical marker key directly from the laboratory database table, accepting a list of canonical lab keys with the same temporal scope options as the report retrieval tool. The set of available canonical keys was determined per patient at runtime and provided to the language model in the prompt to prevent queries for non-existent markers. A default limit of five results per marker was applied.

Four deterministic clinical scoring calculators were provided, each implemented as a stateless function with validated inputs and JSON output. The *ISS* (International Staging System) assigned Stage I when serum *β*_2_-microglobulin was below 3.5 mg/L and serum albumin was at least 3.5 g/dL, Stage III when *β*_2_-microglobulin was 5.5 mg/L or above, and Stage II otherwise. The *R-ISS* (Revised ISS) assigned Stage I when ISS Stage I criteria were met, together with the absence of high-risk cytogenetic abnormalities (del(17p), t(4;14), or t(14;16)) and normal LDH, Stage III when ISS Stage III criteria were met together with either high-risk cytogenetics or elevated LDH, and Stage II otherwise. The *R2-ISS* (Second Revision ISS) incorporated additional risk factors, including the LDH/ULN ratio and 1q gain/amplification status, yielding four stages (I–IV). The *HCT-CI* (Hematopoietic Cell Transplantation Comorbidity Index) was computed from 17 boolean comorbidity flags with weights of 1 (arrhythmia, cardiac disease, inflammatory bowel disease, diabetes requiring medication, cerebrovascular disease, psychiatric disturbance, mild hepatic abnormality, obesity with BMI above 35, and persistent infection), 2 (rheumatologic disease, peptic ulcer, moderate-to-severe renal impairment, and moderate pulmonary disease), or 3 (prior solid tumor, heart valve disease, severe pulmonary disease, and moderate-to-severe hepatic disease), with risk groups defined as low (score 0), intermediate (score 1–2), and high (score ≥3).

**Phase IV: Final answer synthesis**. After tool execution, accumulated context nodes were deduplicated and assigned sequential citation identifiers. The language model received the original question, patient context, a plan execution summary, the formatted context snippets with citation identifiers, the style context from selected skills, the response requirements derived from the question assessment, and the output of the policy engine, where applicable. The policy engine was a deterministic module that ranked retrieved evidence items by temporal authority and recency, resolved contradictions between planned and administered therapies, and produced a structured resolution (select, abstain, or conflict) passed to the final answer signature.

The final answer was required to conform to a two-line format comprising an “Answer”: line containing the schema-aligned value and a “Reasoning”: line containing one to two sentences with inline citation references. If the initial output did not conform to this format, a repair prompt was appended, and the answer was regenerated (up to two attempts). If no valid answer was produced after all attempts, the run was recorded as a pipeline failure. Citation identifiers appearing in the answer were validated against the set of available context nodes, and citations referencing non-existent sources were flagged as potentially hallucinated.

**Decoding and inference settings**. All language models were deployed locally on a single NVIDIA H200 GPU using a vLLM inference server with LiteLLM as the routing layer, providing an OpenAI-compatible API endpoint. The primary model (gpt-oss-120b) was served in mxfp4 precision. Decoding parameters were set to the values recommended by the respective model developers and held constant within each model’s evaluation; no parameter optimization was performed on the evaluation cohort. Run-to-run variability attributable to stochastic decoding was characterized through the ten-run stability analysis (Supplementary Table 4).

#### Comparator configurations

Simple RAG, Iterative RAG, and Full Context are described above. All comparators used the same locally deployed 120-billion-parameter language model (gpt-oss-120b), the same patient record database, and the same structured answer prompt requiring the two-line format. The temperature was set to 0.2 for all comparator configurations. Maximum output tokens were set to 512 for answer generation and 1024 for query generation, where applicable. The embedding model used for dense retrieval was distiluse-base-multilingual-cased-v2, a multilingual sentence transformer producing normalized embeddings for cosine similarity computation.

The LLM Baseline configuration received only the patient identifier, the question text, a reference date, and the answer schema, with no record access or retrieval context of any kind. The system prompt instructed the model to answer based solely on its parametric knowledge, establishing a floor for performance without record access.

Advanced RAG extended Simple RAG with query rewriting into up to eight focused retrieval queries, hybrid fusion of BM25 and dense scores (BM25 built over character trigrams with *k*_1_ = 1.2 and *b* = 0.75, fusion weight *α* = 0.5), and cross-encoder reranking using bge-reranker-v2-m3. The top 20 documents after reranking were packed into the context window, subject to the same 120,000-token budget. This configuration is reported in Supplementary Table 3 but is not included in the main comparator set.

Skill-augmented Iterative RAG (Skill RAG) reused the Iterative RAG pipeline unchanged and prepended the agent’s Phase I skill selection: the same *AssessAndSelectSkills* signature with the same inputs, the same heuristic keyword fallback and always-included base style skill, and the same deterministic policy-skill attachment, the only difference being that no tool list was present in the prompt. The selected modules were rendered with the agent’s own rendering functions into the two context blocks described above and injected at the points given under Agentic system and comparators; policy skills, which the agent passes by identifier to its deterministic policy engine, were rendered as plain-text instructions because no policy engine was available. The selected modules corresponded to ~8000 tokens of skill context, which counted against the same 120,000-token budget as the retrieved documents. A second variant loaded all 42 skill modules unconditionally at the same injection points (approximately 49,000 tokens), mirroring the unfiltered-library ablation of the agent; it was evaluated over five runs and is reported in Supplementary Table 5 only.

### Error classification and citation sufficiency

All patient-question pairs for which the agentic system diverged from expert consensus were classified by severity using a six-category taxonomy aligned with the adjudication categories applied during expert annotation, enabling direct comparison of system error rates with expert disagreement rates at equivalent severity levels. Citation sufficiency, the degree to which retrieved sources supported each answer, was assessed separately.

#### Error classification protocol

Error classification was performed by the senior hematologist (KB) on a single evaluation run with overall concordance of 77.8%, 2.3 percentage points below the ten-run mean of 80.1% and below the lowest of the ten reported runs (78.0%), selected to provide a conservative basis for error rate estimation. All 115 patient-question pairs for which the system output diverged from the adjudicated expert consensus, including list-type responses with partial but incomplete overlap, were submitted for classification.

Classification was performed using a purpose-built review application that presented each case with the question text, answer schema, the adjudicated reference annotation, and the full agent response comprising the extracted answer, the reasoning chain with inline citation identifiers, and all retrieved source documents. Retrieved documents were separated into those cited in the agent’s reasoning and those retrieved but not cited; each could be expanded to display the full report text with the cited passage highlighted. The reviewer additionally had access to the institutional SAP interface to verify claims against the primary clinical record when needed.

Each divergence was classified into exactly one of six categories. Annotation error (category A) covered cases in which the system response was correct and the adjudicated reference was wrong or incomplete. Acceptable or ambiguous (category B) covered cases in which both the system response and the reference were clinically defensible, given the available evidence. Partially correct (category C) covered cases in which the system captured the correct clinical concept, but with incomplete or imprecise details. Clinically insignificant error (category D) covered cases in which the system response was incorrect, but the error would not alter a clinical decision. Clinically significant error (category E) covered cases in which the system response was incorrect in a way that could alter a clinical decision. Pipeline failure (category F) covered cases in which the system did not produce a valid, schema-conformant response within the permitted retry budget. The threshold separating clinically insignificant from clinically significant errors was whether a treating clinician relying on the system response rather than the correct answer would be expected to make a different management decision.

The clinically significant error rate reported in the Results (7.0%, 33 of 469 evaluable pairs) was defined as the proportion of category E errors over all evaluable pairs. Because the classified run had below-average concordance, this rate is a conservative estimate. Error classification was performed by a single reviewer without an independent second review, a limitation acknowledged in the Discussion.

To assess the reproducibility of the primary error classification, a blinded re-annotation sub-study was conducted on a proportional stratified random sample of the 115 divergent patient-question pairs. The pairs were collapsed into three severity strata before sampling, comprising cases classified as annotation error or acceptable and ambiguous (categories A and B), cases classified as partially correct or clinically insignificant error (categories C and D), and cases classified as clinically significant error or pipeline failure (categories E and F). A 40 % proportional stratified random sample was drawn from each stratum (seed = 42), with any rounding remainder assigned to the E+F stratum, yielding *n* = 46 cases. Cases were presented in order of patient and question identifier to an independent rater (JS), blinded to the original classification, who applied the same six-category taxonomy and clinical-significance threshold used in the primary error classification. Agreement is quantified using Cohen’s *κ* for both the collapsed three-stratum scheme and the full six-category scheme, with 95% confidence intervals computed by bootstrap (*N*_boot_ = 10,000).

#### Citation sufficiency assessment

Citation sufficiency was assessed on the same evaluation run used for error classification (overall concordance 77.8%). A stratified sample of 96 agentic system responses was drawn, comprising 16 responses from each of six cells defined by the cross-classification of complexity level (Level 1, Level 2, and Level 3) and concordance status (concordant and discordant), with the constraint that each of the 48 question templates contributed at least one response. Within each cell, responses were drawn randomly from the available pool after satisfying the template coverage constraint.

Assessment was performed by two reviewers (KB and CN), who divided the 96 cases between them and used the same review application and record access as for error classification.

For each response, the reviewer assessed whether the cited sources fully supported the answer on a three-point scale: fully supported (cited evidence clearly and completely justified the answer), partially supported (cited evidence covered some aspects but was incomplete or insufficient), or not supported (cited evidence did not justify the answer). Support was assessed independently of answer correctness. For responses rated as partially or not supported, the reviewer identified whether a relevant document appeared in the retrieved-but-not-cited set, and classified the dominant failure mechanism as one of three types: reasoning failure (the relevant document was retrieved but not used or was misinterpreted during synthesis), retrieval failure (the relevant document existed in the record but was not returned by the retrieval tools), or true knowledge gap (the information required to answer the question was not present in the patient record).

### Statistical analysis

The primary outcome was concordance with expert consensus, defined as the proportion of pairs for which system output matched the adjudicated reference on substantive content, with list-type items scored by entry-level F1. Secondary outcomes were the clinically significant error rate and citation sufficiency. Record-length sensitivity was examined as a pre-specified exploratory analysis (Supplementary Table 9). Reporting followed the TRIPOD + AI guidelines^29^.

#### Scoring and concordance computation

For single-value categorical items, scoring was binary: a score of 1 was assigned if the system output matched the adjudicated reference in substantive content and 0 otherwise. Matching was performed after normalization of both the system output and the reference annotation, including extraction of the answer value from the two-line output format, case-insensitive comparison, whitespace trimming, and removal of formatting artifacts. Format deviations that did not alter substantive content were not penalized. Semantic equivalences were applied where clinically appropriate, for therapy-related questions, “nie verabreicht” (never administered) and “nicht dokumentiert” (not documented) were treated as equivalent when the patient record contained no evidence that the therapy had ever been given.

For list-type items, F1 was computed over the set of response entries against the reference list, with each entry treated as an atomic unit. Precision was the fraction of system entries matching a reference entry, recall was the fraction of reference entries matched by a system entry, and F1 was their harmonic mean. For list-type items preceded by a status field, the status was evaluated separately: an incorrect status (e.g., “Nie verabreicht” when reference entries existed) resulted in a score of 0 for the entire item, regardless of entry-level matching.

Overall concordance was computed as the mean score across all evaluable patient-question pairs, where each pair contributed either a binary score (0 or 1) or a continuous F1 score (0 to 1) depending on the answer schema type. Per-question scores were first averaged across ten independent runs, and overall concordance was computed from these per-question means.

Confidence intervals and pairwise significance tests were computed using a cluster bootstrap procedure. Whole patients were resampled with replacement, retaining all questions per sampled patient to account for within-patient correlation; the concordance statistic was recomputed on each resample over *N*_boot_ = 10,000 iterations. The 2.5th and 97.5th percentiles of the bootstrap distribution defined the 95% confidence interval (two-sided). The two-sided *p* value for pairwise differences was derived as the proportion of bootstrap resamples in which the absolute deviation of the resampled difference from its mean equaled or exceeded the absolute observed difference (shift-to-null method). Bonferroni correction was applied within each stratum using *m* = 6 headline pairwise comparisons, capped at 1.0. Results of all pairwise comparisons are reported in Supplementary Table 3.

## Supplementary information

Supplementary information

## Data Availability

The MIMIC-IV database used for external validation is publicly available under the PhysioNet credentialed data use agreement at https://doi.org/10.13026/1n74-ne17. The in-house TUM longitudinal myeloma dataset cannot be shared publicly owing to patient-privacy regulations and institutional data-governance requirements. Anonymized aggregate results supporting the findings of this study are available from the corresponding author (johannes.moll@tum.de) on reasonable request from the date of publication. Requests will be reviewed by the study team, and a signed data access agreement will be required before release.

## Code availability

The underlying code for this study, comprising the complete agentic system implementation (retrieval tools, the clinical skill library, scoring calculators, and a database ingestion pipeline designed for institutional deployment), is available on GitHub and can be accessed at https://github.com/jomoll/onco-agent.
