# Supplementary material for: Skill-guided agentic reasoning enables longitudinal synthesis of myeloma records at expert-level concordance
Source: NPJ Precis Oncol. 2026 Oct 14;10:389. doi: 10.1038/s41698-026-01733-2 (PMC13669396; doi:10.1038/s41698-026-01733-2)
Supplement: Supplementary file 1 [file 41698_2026_1733_MOESM1_ESM.pdf]

Supplementary Information

Skill-Guided Agentic Reasoning Enables Longitudinal
Synthesis of Myeloma Records at Expert-Level Concordance

Contents

A Supplementary Tables **ii**

Supplementary Table 1: Baseline characteristics of evaluation cohorts ii
Supplementary Table 2: Clinical question bank ii
Supplementary Table 3: Pairwise cluster bootstrap significance tests for concordance differences . vii
Supplementary Table 4: Run-to-run stability across system configurations xi
Supplementary Table 5: System ablations xi
Supplementary Table 6: System performance across language model backbones xi
Supplementary Table 7: Concordance by clinical task category and system configuration xii
Supplementary Table 8: Execution characteristics by complexity level and system configuration . xii
Supplementary Table 9: Patient stratification by record length xiii

B Supplementary Figures **xiv**

Supplementary Figure 1: Accuracy by patient sex, vital status, and age group xiv
Supplementary Figure 2: Accuracy by question template across different systems xv

C Tripod+AI Reporting Checklist **xvi**

A Supplementary Tables

Supplementary Table 1: Baseline characteristics of evaluation cohorts

Demographic and disease characteristics of patients in the primary evaluation (TUM, $n = 100$) and cross-dataset evaluation (MIMIC-IV, $n = 20$) cohorts. Continuous variables are reported as median (IQR), categorical variables as n (%).

**Characteristic**	**TUM** ($n = 100$)	**MIMIC-IV** ($n = 20$)
Age at diagnosis, years	65.1 (57.3–74.0)	66.5 (58.8-77.5)
Sex (male), n (%)	63 (63%)	13 (65%)
Documents per patient	50.5 (31.0–83.0)	29.0 (14.8–61.8)
Laboratory values per patient	1414 (751–2278)	3411 (1989–7411)
Follow-up span (record), years	5.5 (2.7 – 11.0)	3.3 (1.2 – 4.6)

Supplementary Table 2: Clinical question bank

All 48 question templates used for evaluation, grouped by clinical task category and complexity level. Each template includes the question identifier, complexity level, clinical task category, template text with placeholder variables, expected answer format, and scoring method (binary or list-type F1). [DATE] denotes the date of the last available report. ND = not documented.

ID	L	Category	Template text	Answer format	Scoring
Level 1 – Simple					
Q01	1	Single choice	Is the patient receiving lenalidomide on [DATE]?	Yes / No / ND / Unclear	Binary
Q02	1	Single choice	Is the patient receiving bortezomib on [DATE]?	Yes / No / ND / Unclear	Binary
Q03	1	Single choice	Is the patient receiving daratumumab on [DATE]?	Yes / No / ND / Unclear	Binary
Q04	1	Single choice	Is there documented evidence that a whole-body MRI has been performed?	Yes / No / Unclear	Binary
Q05	1	Single choice	Is there documented evidence that a PET-CT has been performed?	Yes / No / Unclear	Binary
Q06	1	Single choice	Is there documented evidence that FISH has been performed?	Yes / No / Unclear	Binary
Q07	1	Single choice	Has the patient received at least one CD38-antibody therapy?	Yes / No / Unclear	Binary
Q08	1	Single choice	Has the patient received at least one IMiD therapy?	Yes / No / Unclear	Binary
Q09	1	Single choice	Has the patient received at least one proteasome inhibitor?	Yes / No / Unclear	Binary
Q10	1	Single choice	Has the patient received an autologous SCT?	Yes / No / Unclear	Binary
Q11	1	Single choice	Has the patient received an allogeneic SCT?	Yes / No / Unclear	Binary
Q12	1	Single choice	Has the patient received CAR-T therapy?	Yes / No / Unclear	Binary
Q13	1	Single choice	Has the patient received BiTE therapy?	Yes / No / Unclear	Binary
Q14	1	Single choice	Has the patient received dialysis?	Yes / No / Unclear	Binary
Q15	1	Single choice	Has the patient been diagnosed with sepsis or septic shock?	Yes / No / Unclear	Binary
Q16	1	Single choice	Has the patient been mechanically ventilated (invasive or non-invasive)?	Yes / No / Unclear	Binary
Q17	1	Single choice	Is renal failure / AKI documented?	Yes / No / ND / Unclear	Binary
Q18	1	Single choice	Is clinically relevant anaemia present on [DATE] (documented or lab-confirmed)?	Yes / No / ND / Unclear	Binary

ID	L	Category	Template text	Answer format	Scoring
Q19	1	Single choice	Has the patient received red blood cell transfusions?	Yes / No / Unclear	Binary
Q20	1	Single choice	Has the patient been diagnosed with pneumonia?	Yes / No / Unclear	Binary
Level 2 – Medium					
Q21	2	Treatment intervals	Which cycle start dates (C*D1) and doses for bortezomib are documented?	$\leq$12$\times$(date; dose; unit)	List-type F1
Q22	2	Treatment intervals	Which cycle start dates (C*D1) and doses for lenalidomide are documented?	$\leq$12$\times$(date; dose; unit)	List-type F1
Q23	2	Treatment intervals	Which melphalan exposures are documented (type, approximate date, dose per episode)?	$\leq$3$\times$(type; date; dose; unit)	List-type F1
Q24	2	Treatment intervals	Which doxorubicin exposures are documented (form, approximate date, cumulative dose per episode)?	$\leq$3$\times$(form; date; dose; unit)	List-type F1
Q25	2	Treatment intervals	In which documented intervals was carfilzomib administered (start–end or ongoing)?	$\leq$3$\times$(start–end / ongoing)	List-type F1
Q26	2	Treatment intervals	In which documented intervals was pomalidomide administered (start–end or ongoing)?	$\leq$3$\times$(start–end / ongoing)	List-type F1
Q27	2	Treatment intervals	In which documented intervals was meropenem administered (start–end or ongoing)?	$\leq$3$\times$(start–end / ongoing)	List-type F1
Q28	2	Single choice	Was toxicity, dose reduction, or discontinuation documented under carfilzomib? If yes, reason?	Yes / No / Unclear + free text	Binary
Q29	2	Single choice	Was toxicity, dose reduction, or discontinuation documented under pomalidomide? If yes, reason?	Yes / No / Unclear + free text	Binary
Q30	2	Single choice	Best documented response under isatuximab + Pom-Dex?	CR/VGPR/PR/SD/PD / Never / ND / Unclear	Binary
Q31	2	Single choice	Best documented response under KRD (carfilzomib–lenalidomide–dexamethasone)?	CR/VGPR/PR/SD/PD / Never / ND / Unclear	Binary
Q32	2	Single choice	Best documented response under the documented first-line therapy?	CR/VGPR/PR/SD/PD / Never / ND / Unclear	Binary

ID	L	Category	Template text	Answer format	Scoring
Q33	2	Single choice	Best documented response after high-dose melphalan?	CR/VGPR/PR/SD/PD / Never / ND / Unclear	Binary
Q34	2	Single choice	Best documented response under BiTE?	CR/VGPR/PR/SD/PD / Never / ND / Unclear	Binary
Q35	2	Single choice	Best documented response under CAR-T?	CR/VGPR/PR/SD/PD / Never / ND / Unclear	Binary
Q36	2	First occurrence	First documented CT report describing new osteolytic lesions?	Date / ND / Unclear	Binary
Q37	2	First occurrence	First documented episode of renal failure or dialysis-requiring AKI?	Date / ND / Unclear	Binary
Q38	2	Staging	ISS stage on [DATE] calculated from albumin and β_2M (last labs $\leq$90 days prior)?	Score (I/II/III) + date + source	Binary
Level 3 – Complex					
Q39	3	Staging	ECOG score on [DATE] (last documented $\leq$180 days, else inferred from explicit functional description)?	Score (0–4) + date + source	Binary
Q40	3	Staging	HCT-CI score on [DATE] (last documented $\leq$365 days, else derived from comorbidities)?	Score + date + source	Binary
Q41	3	Staging	R-ISS stage on [DATE] (labs $\leq$90 days; cytogenetics from last available report)?	Stage (I/II/III) + date + source	Binary
Q42	3	Staging	R2-ISS stage on [DATE] (labs $\leq$90 days; cytogenetics incl. 1q status from last available report)?	Stage (I/II/III/IV) + date + source	Binary
Q43	3	Single choice	Is the patient triple-class refractory on [DATE]? (with documented justification)	Yes / No / Unclear + free text	Binary
Q44	3	Single choice	Is the patient quadruple-class refractory on [DATE]? (with documented justification)	Yes / No / Unclear + free text	Binary
Q45	3	Single choice	Is the patient eligible for ASCT on [DATE] based on age, comorbidities, performance status, and organ function?	Yes / No / Unclear + free text	Binary

ID	L	Category	Template text	Answer format	Scoring
Q46	3	Single choice	Which therapy achieved the best documented response up to [DATE] (per IMWG criteria)?	Free text (therapy) + CR/VGPR/PR/SD/PD / ND / Unclear	Binary
Q47	3	Single choice	Which risks currently dominate: disease progression or therapy toxicity?	Progression / Toxicity / Both / Unclear + free text	Binary
Q48	3	Eligibility	Which BCMA-CAR-T eligibility criteria are met / not met / missing on [DATE]? Is the patient eligible overall?	Criteria table (met/not met/missing) + Yes / No / Unclear	Binary

Supplementary Table 3: Pairwise cluster bootstrap significance tests for concordance differences

All pairwise comparisons between system configurations, overall, stratified by complexity level, and stratified by record-length bin (Supplementary Table 9). Skill RAG denotes Iterative RAG augmented with the agent's clinical skill library. Differences are reported as System A minus System B in percentage points; a negative value indicates that System B outperformed System A. Confidence intervals and p-values were obtained by cluster bootstrap with $N_{\text{boot}} = 10{,}000$ resamples, resampling whole patients with replacement and retaining all questions per sampled patient to account for within-patient correlation. The N column reports the number of patients contributing to each stratum. Bonferroni-corrected p-values were computed by multiplying raw p-values by the number of pairwise comparisons within each stratum (6 headline comparisons), capped at 1.0. Significance codes reflect Bonferroni-corrected p-values: $^{***}p < 0.001$; $^{**}p < 0.01$; $^{*}p < 0.05$; ns $p \geq 0.05$.

Subset	Comparison (A vs B)	N patients	A−B (pp)	95% CI	Raw p	Bonf. p	
Overall	Baseline vs Simple RAG	100	−70.22	[−74.00, −66.44]	<0.001	<0.001	***
Overall	Baseline vs Advanced RAG	100	−74.81	[−77.95, −71.59]	<0.001	<0.001	***
Overall	Baseline vs Iterative RAG	100	−74.42	[−77.64, −71.11]	<0.001	<0.001	***
Overall	Baseline vs Full Context	100	−74.55	[−77.93, −71.18]	<0.001	<0.001	***
Overall	Baseline vs Skill RAG	100	−77.72	[−81.14, −74.27]	<0.001	<0.001	***
Overall	Baseline vs Agentic System	100	−78.85	[−82.03, −75.55]	<0.001	<0.001	***
Overall	Simple RAG vs Advanced RAG	100	−4.58	[−6.96, −2.30]	<0.001	<0.001	***
Overall	Simple RAG vs Iterative RAG	100	−4.19	[−7.01, −1.42]	0.0027	0.0162	*
Overall	Simple RAG vs Full Context	100	−4.33	[−6.79, −1.89]	<0.001	0.003	**
Overall	Simple RAG vs Skill RAG	100	−7.50	[−9.96, −5.11]	<0.001	<0.001	***
Overall	Simple RAG vs Agentic System	100	−8.63	[−11.86, −5.34]	<0.001	<0.001	***
Overall	Advanced RAG vs Iterative RAG	100	+0.39	[−1.37, +2.20]	0.6685	1.000	ns
Overall	Advanced RAG vs Full Context	100	+0.26	[−1.70, +2.17]	0.796	1.000	ns
Overall	Advanced RAG vs Skill RAG	100	−2.92	[−4.60, −1.24]	<0.001	0.0036	**
Overall	Advanced RAG vs Agentic System	100	−4.05	[−6.53, −1.63]	0.0012	0.0072	**
Overall	Iterative RAG vs Full Context	100	−0.13	[−2.39, +2.08]	0.9057	1.000	ns
Overall	Iterative RAG vs Skill RAG	100	−3.31	[−5.27, −1.46]	<0.001	0.0024	**
Overall	Iterative RAG vs Agentic System	100	−4.43	[−7.31, −1.68]	0.0024	0.0144	*
Overall	Full Context vs Skill RAG	100	−3.17	[−5.45, −0.95]	0.0068	0.0408	*
Overall	Full Context vs Agentic System	100	−4.30	[−6.76, −1.88]	<0.001	0.0042	**
Overall	Skill RAG vs Agentic System	100	−1.13	[−3.55, +1.23]	0.3576	1.000	ns
Level 1	Baseline vs Simple RAG	100	−78.90	[−83.55, −73.95]	<0.001	<0.001	***
Level 1	Baseline vs Advanced RAG	100	−84.50	[−88.65, −80.05]	<0.001	<0.001	***
Level 1	Baseline vs Iterative RAG	100	−81.65	[−86.05, −76.85]	<0.001	<0.001	***
Level 1	Baseline vs Full Context	100	−84.90	[−89.00, −80.45]	<0.001	<0.001	***
Level 1	Baseline vs Skill RAG	100	−87.85	[−91.95, −83.40]	<0.001	<0.001	***
Level 1	Baseline vs Agentic System	100	−86.35	[−90.55, −81.75]	<0.001	<0.001	***
Level 1	Simple RAG vs Advanced RAG	100	−5.60	[−9.25, −2.10]	0.0026	0.0156	*
Level 1	Simple RAG vs Iterative RAG	100	−2.75	[−6.90, +1.35]	0.1869	1.000	ns
Level 1	Simple RAG vs Full Context	100	−6.00	[−9.60, −2.65]	0.0019	0.0114	*
Level 1	Simple RAG vs Skill RAG	100	−8.95	[−13.05, −5.05]	<0.001	<0.001	***
Level 1	Simple RAG vs Agentic System	100	−7.45	[−12.15, −2.75]	0.0032	0.0192	*
Level 1	Advanced RAG vs Iterative RAG	100	+2.85	[+0.40, +5.45]	0.0275	0.165	ns
Level 1	Advanced RAG vs Full Context	100	−0.40	[−3.35, +2.50]	0.7861	1.000	ns
Level 1	Advanced RAG vs Skill RAG	100	−3.35	[−5.45, −1.40]	0.0015	0.009	**
Level 1	Advanced RAG vs Agentic System	100	−1.85	[−5.40, +1.70]	0.302	1.000	ns
Level 1	Iterative RAG vs Full Context	100	−3.25	[−6.60, −0.00]	0.052	0.312	ns
Level 1	Iterative RAG vs Skill RAG	100	−6.20	[−9.30, −3.45]	<0.001	0.0012	**
Level 1	Iterative RAG vs Agentic System	100	−4.70	[−8.95, −0.50]	0.0295	0.177	ns
Level 1	Full Context vs Skill RAG	100	−2.95	[−6.00, +0.10]	0.0569	0.3414	ns
Level 1	Full Context vs Agentic System	100	−1.45	[−4.70, +1.80]	0.3873	1.000	ns
Level 1	Skill RAG vs Agentic System	100	+1.50	[−1.50, +4.65]	0.3415	1.000	ns

Supplementary Table 3 (continued): Pairwise cluster bootstrap significance tests for concordance differences

Subset	Comparison (A vs B)	N patients	A−B (pp)	95% CI	Raw p	Bonf. p	
Level 2	Baseline vs Simple RAG	99	−72.65	[−78.74, −66.45]	<0.001	<0.001	***
Level 2	Baseline vs Advanced RAG	99	−75.89	[−81.31, −70.36]	<0.001	<0.001	***
Level 2	Baseline vs Iterative RAG	99	−76.60	[−81.78, −71.08]	<0.001	<0.001	***
Level 2	Baseline vs Full Context	99	−73.88	[−79.80, −67.71]	<0.001	<0.001	***
Level 2	Baseline vs Skill RAG	99	−75.99	[−81.79, −70.00]	<0.001	<0.001	***
Level 2	Baseline vs Agentic System	99	−77.89	[−83.41, −72.20]	<0.001	<0.001	***
Level 2	Simple RAG vs Advanced RAG	99	−3.24	[−7.10, +0.50]	0.094	0.564	ns
Level 2	Simple RAG vs Iterative RAG	99	−3.95	[−8.18, +0.16]	0.065	0.39	ns
Level 2	Simple RAG vs Full Context	99	−1.23	[−5.11, +2.63]	0.5294	1.000	ns
Level 2	Simple RAG vs Skill RAG	99	−3.34	[−7.37, +0.60]	0.0991	0.5946	ns
Level 2	Simple RAG vs Agentic System	99	−5.23	[−8.82, −1.93]	0.0028	0.0168	*
Level 2	Advanced RAG vs Iterative RAG	99	−0.72	[−3.24, +1.76]	0.5722	1.000	ns
Level 2	Advanced RAG vs Full Context	99	+2.01	[−1.31, +5.58]	0.2497	1.000	ns
Level 2	Advanced RAG vs Skill RAG	99	−0.10	[−2.42, +2.11]	0.9236	1.000	ns
Level 2	Advanced RAG vs Agentic System	99	−2.00	[−4.85, +0.85]	0.1649	0.9894	ns
Level 2	Iterative RAG vs Full Context	99	+2.72	[−1.06, +6.73]	0.1688	1.000	ns
Level 2	Iterative RAG vs Skill RAG	99	+0.61	[−1.81, +3.21]	0.6278	1.000	ns
Level 2	Iterative RAG vs Agentic System	99	−1.28	[−4.66, +2.15]	0.4461	1.000	ns
Level 2	Full Context vs Skill RAG	99	−2.11	[−6.38, +1.94]	0.3151	1.000	ns
Level 2	Full Context vs Agentic System	99	−4.01	[−8.00, −0.30]	0.0407	0.2442	ns
Level 2	Skill RAG vs Agentic System	99	−1.89	[−5.05, +1.31]	0.2538	1.000	ns
Level 3	Baseline vs Simple RAG	90	−46.11	[−54.67, −37.44]	<0.001	<0.001	***
Level 3	Baseline vs Advanced RAG	90	−51.11	[−58.89, −43.11]	<0.001	<0.001	***
Level 3	Baseline vs Iterative RAG	90	−54.00	[−62.56, −45.11]	<0.001	<0.001	***
Level 3	Baseline vs Full Context	90	−52.89	[−61.44, −44.22]	<0.001	<0.001	***
Level 3	Baseline vs Skill RAG	90	−58.67	[−67.11, −50.00]	<0.001	<0.001	***
Level 3	Baseline vs Agentic System	90	−64.11	[−72.78, −55.00]	<0.001	<0.001	***
Level 3	Simple RAG vs Advanced RAG	90	−5.00	[−9.56, −0.67]	0.0291	0.1746	ns
Level 3	Simple RAG vs Iterative RAG	90	−7.89	[−14.00, −2.11]	0.0095	0.057	ns
Level 3	Simple RAG vs Full Context	90	−6.78	[−13.44, −0.11]	0.0462	0.2772	ns
Level 3	Simple RAG vs Skill RAG	90	−12.56	[−18.11, −7.44]	<0.001	<0.001	***
Level 3	Simple RAG vs Agentic System	90	−18.00	[−26.78, −9.33]	<0.001	<0.001	***
Level 3	Advanced RAG vs Iterative RAG	90	−2.89	[−7.33, +1.34]	0.1971	1.000	ns
Level 3	Advanced RAG vs Full Context	90	−1.78	[−6.44, +3.00]	0.4632	1.000	ns
Level 3	Advanced RAG vs Skill RAG	90	−7.56	[−13.22, −2.11]	0.0081	0.0486	*
Level 3	Advanced RAG vs Agentic System	90	−13.00	[−20.44, −5.78]	<0.001	0.0012	**
Level 3	Iterative RAG vs Full Context	90	+1.11	[−3.89, +6.22]	0.6685	1.000	ns
Level 3	Iterative RAG vs Skill RAG	90	−4.67	[−9.89, +0.22]	0.0685	0.411	ns
Level 3	Iterative RAG vs Agentic System	90	−10.11	[−17.11, −3.33]	0.0044	0.0264	*
Level 3	Full Context vs Skill RAG	90	−5.78	[−12.67, +0.56]	0.084	0.504	ns
Level 3	Full Context vs Agentic System	90	−11.22	[−18.11, −4.78]	0.0012	0.0072	**
Level 3	Skill RAG vs Agentic System	90	−5.44	[−12.00, +1.11]	0.1091	0.6546	ns

Supplementary Table 3 (continued): Pairwise cluster bootstrap significance tests for concordance differences

Subset	Comparison (A vs B)	N patients	A−B (pp)	95% CI	Raw p	Bonf. p	
Q1 (≤127k)	Simple RAG vs Iterative RAG	33	−1.48	[−6.47, +3.21]	0.545	1.000	ns
Q1 (≤127k)	Simple RAG vs Full Context	33	−3.48	[−8.37, +1.12]	0.1529	0.9174	ns
Q1 (≤127k)	Simple RAG vs Skill RAG	33	−6.06	[−10.74, −1.70]	0.0091	0.0546	ns
Q1 (≤127k)	Simple RAG vs Agentic System	33	−4.45	[−10.27, +1.23]	0.1318	0.7908	ns
Q1 (≤127k)	Iterative RAG vs Full Context	33	−2.00	[−4.60, +0.63]	0.1388	0.8328	ns
Q1 (≤127k)	Iterative RAG vs Skill RAG	33	−4.58	[−7.97, −1.29]	0.0077	0.0462	*
Q1 (≤127k)	Iterative RAG vs Agentic System	33	−2.97	[−7.21, +1.49]	0.1764	1.000	ns
Q1 (≤127k)	Full Context vs Skill RAG	33	−2.58	[−6.73, +1.46]	0.2123	1.000	ns
Q1 (≤127k)	Full Context vs Agentic System	33	−0.97	[−4.68, +2.77]	0.6169	1.000	ns
Q1 (≤127k)	Skill RAG vs Agentic System	33	+1.61	[−1.57, +4.90]	0.331	1.000	ns
Q2 (127k–282k)	Simple RAG vs Iterative RAG	34	−4.21	[−8.32, −0.11]	0.0434	0.2604	ns
Q2 (127k–282k)	Simple RAG vs Full Context	34	−3.85	[−7.00, −0.52]	0.0191	0.1146	ns
Q2 (127k–282k)	Simple RAG vs Skill RAG	34	−6.66	[−10.26, −3.22]	<0.001	<0.001	***
Q2 (127k–282k)	Simple RAG vs Agentic System	34	−9.45	[−15.11, −4.10]	<0.001	0.0036	**
Q2 (127k–282k)	Iterative RAG vs Full Context	34	+0.36	[−4.39, +5.03]	0.8874	1.000	ns
Q2 (127k–282k)	Iterative RAG vs Skill RAG	34	−2.44	[−6.18, +0.80]	0.1591	0.9546	ns
Q2 (127k–282k)	Iterative RAG vs Agentic System	34	−5.24	[−11.26, +0.55]	0.0839	0.5034	ns
Q2 (127k–282k)	Full Context vs Skill RAG	34	−2.80	[−6.52, +0.89]	0.1346	0.8076	ns
Q2 (127k–282k)	Full Context vs Agentic System	34	−5.60	[−10.30, −0.96]	0.0192	0.1152	ns
Q2 (127k–282k)	Skill RAG vs Agentic System	34	−2.80	[−7.79, +2.01]	0.2614	1.000	ns
Q3 (282k–541k)	Simple RAG vs Iterative RAG	23	−3.94	[−10.50, +2.04]	0.2223	1.000	ns
Q3 (282k–541k)	Simple RAG vs Full Context	23	−3.60	[−8.18, +0.93]	0.1214	0.7284	ns
Q3 (282k–541k)	Simple RAG vs Skill RAG	23	−7.47	[−12.94, −2.85]	0.0051	0.0306	*
Q3 (282k–541k)	Simple RAG vs Agentic System	23	−6.08	[−11.48, −1.11]	0.0233	0.1398	ns
Q3 (282k–541k)	Iterative RAG vs Full Context	23	+0.34	[−4.10, +4.91]	0.8854	1.000	ns
Q3 (282k–541k)	Iterative RAG vs Skill RAG	23	−3.53	[−7.13, −0.18]	0.0468	0.2808	ns
Q3 (282k–541k)	Iterative RAG vs Agentic System	23	−2.15	[−7.12, +2.74]	0.3979	1.000	ns
Q3 (282k–541k)	Full Context vs Skill RAG	23	−3.87	[−8.56, +0.47]	0.0963	0.5778	ns
Q3 (282k–541k)	Full Context vs Agentic System	23	−2.48	[−6.89, +1.76]	0.2641	1.000	ns
Q3 (282k–541k)	Skill RAG vs Agentic System	23	+1.39	[−3.14, +5.73]	0.5378	1.000	ns
Q4 (>541k)	Simple RAG vs Iterative RAG	10	−13.67	[−23.03, −3.70]	0.0041	0.0246	*
Q4 (>541k)	Simple RAG vs Full Context	10	−10.40	[−19.64, −1.43]	0.0237	0.1422	ns
Q4 (>541k)	Simple RAG vs Skill RAG	10	−15.16	[−22.90, −7.99]	<0.001	<0.001	***
Q4 (>541k)	Simple RAG vs Agentic System	10	−25.48	[−34.06, −16.10]	<0.001	<0.001	***
Q4 (>541k)	Iterative RAG vs Full Context	10	+3.28	[−3.87, +11.26]	0.4044	1.000	ns
Q4 (>541k)	Iterative RAG vs Skill RAG	10	−1.49	[−8.23, +5.36]	0.6627	1.000	ns
Q4 (>541k)	Iterative RAG vs Agentic System	10	−11.80	[−18.73, −5.07]	<0.001	0.003	**
Q4 (>541k)	Full Context vs Skill RAG	10	−4.77	[−13.21, +2.71]	0.2447	1.000	ns
Q4 (>541k)	Full Context vs Agentic System	10	−15.08	[−21.67, −9.36]	<0.001	<0.001	***
Q4 (>541k)	Skill RAG vs Agentic System	10	−10.31	[−17.49, −4.17]	0.0029	0.0174	*

Supplementary Table 4: Run-to-run stability across system configurations

Concordance estimates across ten independent evaluation runs for each system configuration on the primary evaluation cohort (TUM, $n = 469$). Results are reported as mean ± standard deviation across runs, overall and stratified by complexity level. Individual run results are listed to characterise the distribution.

System	Overall	Level 1	Level 2	Level 3
Baseline	1.3 ± 0.5%	0.1 ± 0.2%	1.8 ± 1.0%	2.8 ± 1.1%
Simple RAG System	71.5 ± 0.9%	79.0 ± 1.7%	74.4 ± 0.9%	48.9 ± 2.7%
Advanced RAG System	76.1 ± 1.7%	84.7 ± 1.5%	77.7 ± 1.3%	53.9 ± 4.5%
Iterative RAG System	75.7 ± 3.3%	81.8 ± 5.7%	78.4 ± 2.3%	56.8 ± 2.8%
Full Context	75.8 ± 0.5%	85.0 ± 1.0%	75.7 ± 1.1%	55.7 ± 1.8%
Skill RAG System	79.0 ± 1.5%	88.0 ± 2.7%	77.8 ± 0.8%	61.4 ± 2.4%
Agentic System	80.1 ± 1.4%	86.5 ± 1.5%	79.7 ± 0.9%	66.9 ± 3.6%

Supplementary Table 5: System ablations

Concordance across ablated system configurations on the primary evaluation cohort (TUM, $n = 469$). Values are mean concordance (%) across the number of independent runs indicated, overall and stratified by complexity level. Each row removes or replaces one or more components of the full agentic system. The unfiltered skill library condition loads all 42 skill modules unconditionally rather than selecting by query content. The lower block reports Iterative RAG supplied with the same skill library, either selected at runtime (Skill RAG, ten runs) or loaded unconditionally (five runs), completing a two-by-two of architecture by skill delivery. Ablation rows were obtained in a separate series of runs whose standard-configuration mean was 79.6% (the ten reported runs of the standard configuration average 80.1%, Supplementary Table 4). Configurations without a skill library use the same fixed baseline prompt as the comparator systems, without the agent-specific reasoning skills.

Configuration	Overall	Level 1	Level 2	Level 3
Standard configuration (ablation series)	79.6%	86.2%	79.5%	65.1%
No deterministic clinical scoring tools	79.6%	85.1%	80.0%	66.7%
No type and date filters in retrieval tools	79.5%	87.7%	78.1%	63.8%
Full skill library, unfiltered	79.3%	87.7%	78.7%	62.0%
No structured memory state	79.2%	85.9%	79.0%	64.9%
Reactive tool selection (no pre-planned use)	79.2%	86.6%	78.7%	63.8%
No skill library	76.6%	86.0%	77.9%	53.3%
Iterative RAG with the agent's skill library (no agentic components)				
Iterative RAG, no skill library	75.7%	81.8%	78.4%	56.8%
Iterative RAG, selected skill library (Skill RAG)	79.0%	88.0%	77.8%	61.4%
Iterative RAG, full skill library, unfiltered	79.6%	88.8%	77.8%	62.9%

Supplementary Table 6: System performance across language model backbones

All four system configurations were evaluated under each backbone model on the primary evaluation cohort (469 patient-question pairs, TUM). Each model was deployed locally via vLLM using the inference settings

recommended by the respective developer. Weight quantization reflects the precision of the loaded model checkpoint (MXFP4 or FP8), Qwen3-Next-80B-A3B-Instruct additionally used an FP8 KV cache. Overall concordance (%) is reported as mean across ten runs. The model used in the primary analysis is indicated (*).

Model	Params	Quantization	Simple RAG	Iterative RAG	Full Context	Agentic
gpt-oss-120b*	120B	MXFP4	71.5%	75.7%	75.8%	80.1%
GLM-4.5-Air	110B	FP8	64.9%	68.2%	72.0%	77.9%
Qwen3-Next-80B-A3B-Instruct	81B	FP8	75.0%	76.5%	76.1%	76.8%
gemma-4-31B-it	33B	FP8	74.7%	82.4%	81.3%	81.6%
gpt-oss-20b	21B	MXFP4	67.9%	72.1%	68.4%	77.4%

Supplementary Table 7: Concordance by clinical task category and system configuration

Concordance stratified by clinical task category (single choice, treatment intervals, first occurrence, staging, eligibility) for each system configuration on the primary evaluation cohort (TUM, $n = 469$). Values are mean concordance across ten independent runs with 95% bootstrap confidence intervals (cluster bootstrap, 10,000 resamples, patient-level resampling). Category sample sizes: single choice $n = 326$, treatment intervals $n = 70$, first occurrence $n = 20$, staging $n = 45$, eligibility $n = 8$. Results for the eligibility category are based on a small sample and should be considered hypothesis-generating only.

System	Single choice	Treatment intervals	First occurrence	Staging	Eligibility
Baseline	0.1 [0.0–0.2]	2.6 [1.7–3.6]	0.0 [0.0–0.0]	8.4 [6.2–10.9]	0.0 [0.0–0.0]
Simple RAG	76.9 [76.3–77.5]	74.2 [73.0–75.2]	38.0 [35.5–40.5]	55.6 [54.7–56.4]	1.2 [0.0–3.8]
Advanced RAG	81.8 [81.0–82.8]	74.6 [73.5–75.6]	52.5 [47.5–57.0]	56.4 [55.3–57.6]	25.0 [16.2–33.8]
Iterative RAG	80.9 [79.1–82.1]	74.6 [73.0–76.0]	52.0 [48.5–55.5]	55.3 [53.3–57.1]	32.5 [26.2–40.0]
Full Context	83.5 [82.9–84.0]	70.9 [70.1–71.7]	40.0 [37.5–42.5]	53.6 [52.0–55.3]	21.2 [13.8–28.7]
Skill RAG	86.0 [82.5–89.2]	74.2 [63.8–84.4]	54.0 [34.5–73.0]	57.3 [43.0–71.1]	20.0 [7.5–32.5]
Agentic System	87.0 [83.6–90.1]	75.7 [66.2–84.8]	50.0 [31.0–70.0]	53.3 [39.5–66.5]	65.0 [30.0–90.0]

Supplementary Table 8: Execution characteristics by complexity level and system configuration

Wall-clock time per question on the primary evaluation cohort (TUM, n = 469 patient-question pairs from 100 patients), stratified by complexity level and system configuration. Unless otherwise indicated, all values are median (IQR) across all patient-question pairs from a single-concurrency timing run on a single NVIDIA H200 GPU. Skills selected, tool calls, and document retrieval metrics are reported for the agentic system only.

Metric	Level 1 ($n = 200$)	Level 2 ($n = 179$)	Level 3 ($n = 90$)	Overall ($n = 469$)
Agentic system				
Skills selected per question	6.0 (6.0–7.0)	6.0 (6.0–7.0)	8.0 (7.0–9.0)	7.0 (6.0–7.0)
Tool calls per question	2.0 (1.0–3.0)	3.0 (2.0–4.0)	4.0 (3.0–4.0)	3.0 (2.0–4.0)
Documents retrieved	15.0 (7.5–26.0)	27.0 (14.0–49.0)	27.0 (16.0–46.0)	20.0 (10.0–39.0)
Documents cited	2.0 (2.0–2.0)	2.0 (2.0–2.0)	2.0 (2.0–4.0)	2.0 (2.0–2.0)
of which $\geq$3 documents (%)	5.9	9.5	47.4	15.2
Wall-clock time per question, s	18.5 (16.7–20.6)	21.3 (19.4–23.8)	28.7 (26.3–34.2)	20.9 (18.2–25.2)
Iterative RAG				
Wall-clock time per question, s	6.0 (5.0–7.3)	6.4 (5.1–8.6)	12.3 (7.2–23.4)	6.6 (5.3–9.5)
Full Context				
Wall-clock time per question, s	4.7 (3.8–5.6)	5.0 (4.0–6.0)	6.0 (5.1–6.9)	5.0 (4.0–6.0)
Simple RAG				
Wall-clock time per question, s	2.2 (1.8–2.8)	2.2 (1.8–2.8)	2.7 (2.2–3.1)	2.3 (1.8–2.9)

Supplementary Table 9: Patient stratification by record length

Patients were stratified by total clinical record length (character count of the full concatenated record) into four bins for the context-length sensitivity analysis. Three bins span the lower 90th percentile of the distribution (Q1–Q3), defined at the 33rd and 67th percentiles, the fourth bin (Q4) comprises the top decile. Evaluable pairs are summed across all complexity levels.

Bin	Character range	Patients (n)	Evaluable pairs (n)
Q1 ($\leq$p33)	$\leq$127k	33	155
Q2 (p33–p67)	127k–282k	34	160
Q3 (p67–p90)	282k–541k	23	107
Q4 ($>$p90)	$>$541k	10	47
Total	$\leq$1,076k	100	469

B Supplementary Figures

Supplementary Figure 1: Accuracy by patient sex, vital status, and age group

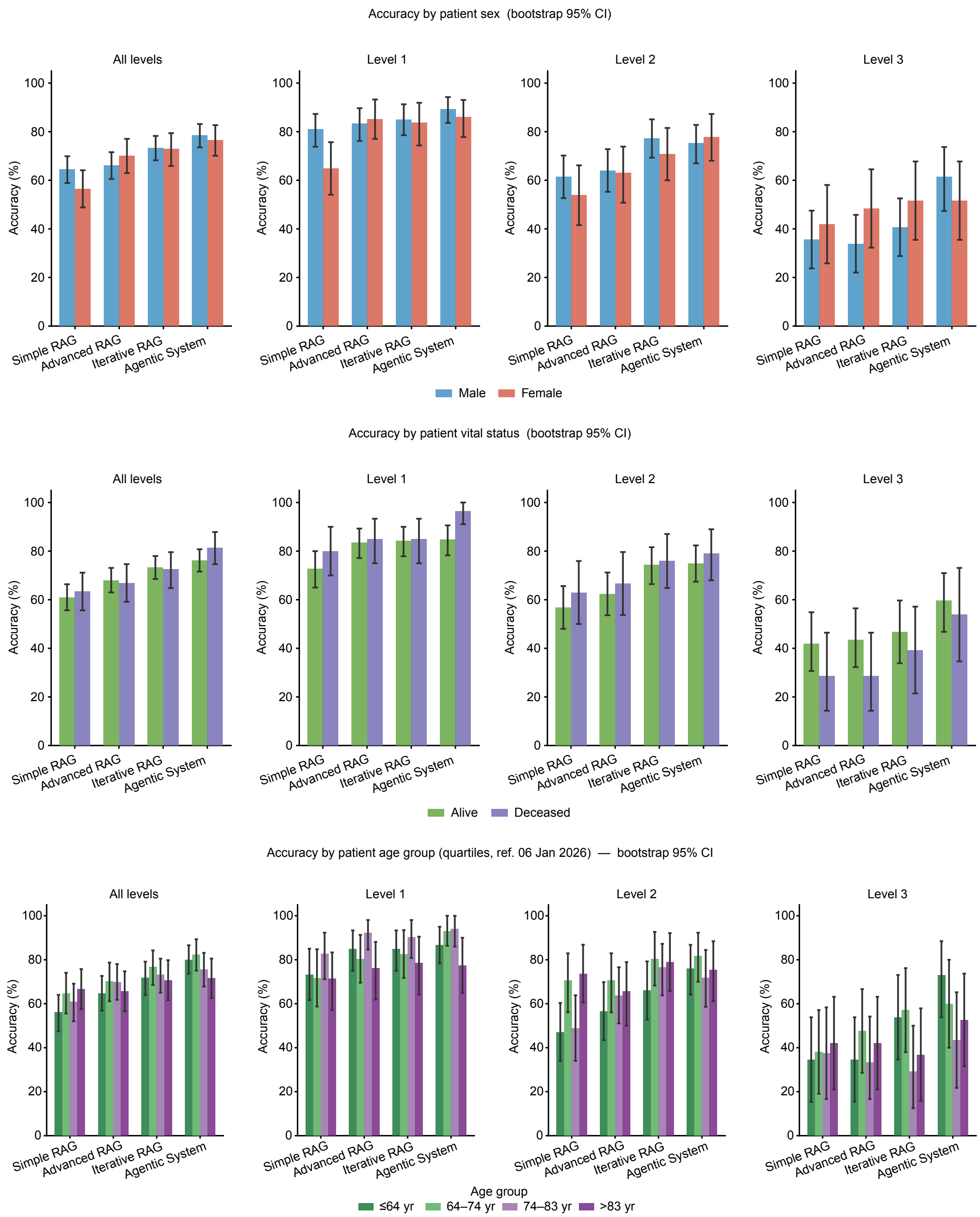

Supplementary Figure 1. Concordance with expert consensus stratified by patient sex (male, female), vital status (alive, deceased), and age group at the reference date (quartiles: $\leq$64, 64–74, 74–83, $>$83 years), shown overall and by complexity level for all four system configurations. Bootstrap 95% confidence intervals were computed by cluster bootstrap resampling at the patient level ($N_{\text{boot}} = 10{,}000$).

Supplementary Figure 2: Accuracy by question template across different systems

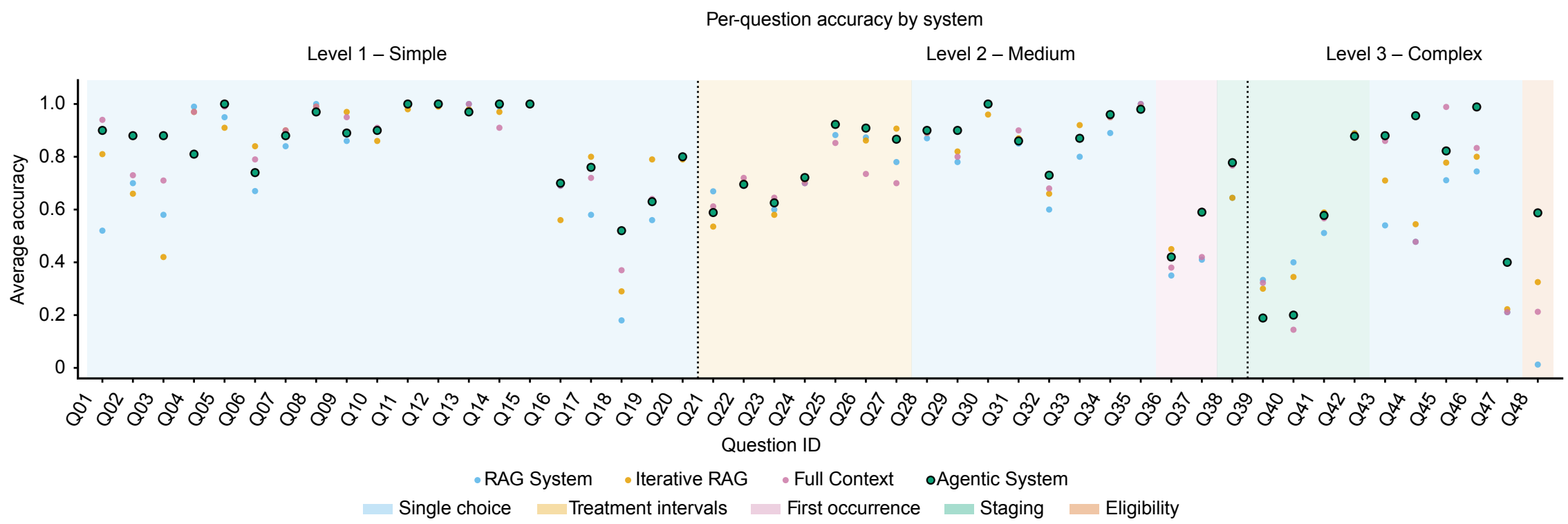

Supplementary Figure 2. Per-question concordance averaged across ten independent evaluation runs for each of the 48 clinical question templates, stratified by system configuration and clinical task category (single choice, treatment intervals, first occurrence, staging, eligibility). Templates are ordered by complexity level (Level 1, Level 2, Level 3) and grouped by task category within each level.

C Tripod+AI Reporting Checklist

Version: 11-January-2024

Section/Topic	Item	Development / evaluation[1]	Checklist item	Reported on page
**TITLE**				
Title	1	D;E	Identify the study as developing or evaluating the performance of a multivariable prediction model, the target population, and the outcome to be predicted	1
**ABSTRACT**				
Abstract	2	D;E	See TRIPOD+AI for Abstracts checklist	1-2
**INTRODUCTION**				
Background	3a	D;E	Explain the healthcare context (including whether diagnostic or prognostic) and rationale for developing or evaluating the prediction model, including references to existing models	2
	3b	D;E	Describe the target population and the intended purpose of the prediction model in the context of the care pathway, including its intended users (e.g., healthcare professionals, patients, public)	2
	3c	D;E	Describe any known health inequalities between sociodemographic groups	Suppl.Fig. 1
Objectives	4	D;E	Specify the study objectives, including whether the study describes the development or validation of a prediction model (or both)	2-3
**METHODS**				
Data	5a	D;E	Describe the sources of data separately for the development and evaluation datasets (e.g., randomised trial, cohort, routine care or registry data), the rationale for using these data, and representativeness of the data	8
	5b	D;E	Specify the dates of the collected participant data, including start and end of participant accrual; and, if applicable, end of follow-up	8
Participants	6a	D;E	Specify key elements of the study setting (e.g., primary care, secondary care, general population) including the number and location of centres	8
	6b	D;E	Describe the eligibility criteria for study participants	8, Figure 3
	6c	D;E	Give details of any treatments received, and how they were handled during model development or evaluation, if relevant	N/A
Data preparation	7	D;E	Describe any data pre-processing and quality checking, including whether this was similar across relevant sociodemographic groups	9-10
Outcome	8a	D;E	Clearly define the outcome that is being predicted and the time horizon, including how and when assessed, the rationale for choosing this outcome, and whether the method of outcome assessment is consistent across sociodemographic groups	10-11, 19
	8b	D;E	If outcome assessment requires subjective interpretation, describe the qualifications and demographic characteristics of the outcome assessors	11
	8c	D;E	Report any actions to blind assessment of the outcome to be predicted	11-12
Predictors	9a	D	Describe the choice of initial predictors (e.g., literature, previous models, all available predictors) and any pre-selection of predictors before model building	N/A
	9b	D;E	Clearly define all predictors, including how and when they were measured (and any actions to blind assessment of predictors for the outcome and other predictors)	N/A
	9c	D;E	If predictor measurement requires subjective interpretation, describe the qualifications and demographic characteristics of the predictor assessors	N/A
Sample size	10	D;E	Explain how the study size was arrived at (separately for development and evaluation), and justify that the study size was sufficient to answer the research question. Include details of any sample size calculation	10-11, Figure 3
Missing data	11	D;E	Describe how missing data were handled. Provide reasons for omitting any data	10-11
Analytical methods	12a	D	Describe how the data were used (e.g., for development and evaluation of model performance) in the analysis, including whether the data were partitioned, considering any sample size requirements	10-11
	12b	D	Depending on the type of model, describe how predictors were handled in the analyses (functional form, rescaling, transformation, or any standardisation).	N/A
	12c	D	Specify the type of model, rationale[2], all model-building steps, including any hyperparameter tuning, and method for internal validation	13-16
	12d	D;E	Describe if and how any heterogeneity in estimates of model parameter values and model performance was handled and quantified across clusters (e.g., hospitals, countries). See TRIPOD -Cluster for additional considerations[3]	19
	12e	D;E	Specify all measures and plots used (and their rationale) to evaluate model performance (e.g., discrimination, calibration, clinical utility) and, if relevant, to compare multiple models	19
	12f	E	Describe any model updating (e.g., recalibration) arising from the model evaluation, either overall or for particular sociodemographic groups or settings	N/A
	12g	E	For model evaluation, describe how the model predictions were calculated (e.g., formula, code, object, application programming interface)	16-17
Class imbalance	13	D;E	If class imbalance methods were used, state why and how this was done, and any subsequent methods to recalibrate the model or the model predictions	N/A
Fairness	14	D;E	Describe any approaches that were used to address model fairness and their rationale	N/A
Model output	15	D	Specify the output of the prediction model (e.g., probabilities, classification). Provide details and rationale for any classification and how the thresholds were identified	16

[1] D=items relevant only to the development of a prediction model; E=items relating solely to the evaluation of a prediction model; D;E=items applicable to both the development and evaluation of a prediction model

[2] Separately for all model building approaches.

[3] TRIPOD-Cluster is a checklist of reporting recommendations for studies developing or validating models that explicitly account for clustering or explore heterogeneity in model performance (eg, at different hospitals or centres). Debray et al, BMJ 2023; 380: e071018 [DOI: 10.1136/bmj-2022-071018]

Training versus evaluation	16	D;E	Identify any differences between the development and evaluation data in healthcare setting, eligibility criteria, outcome, and predictors	8, Figure 2, Figure 3
Ethical approval	17	D;E	Name the institutional research board or ethics committee that approved the study and describe the participant-informed consent or the ethics committee waiver of informed consent	8
**OPEN SCIENCE**				
Funding	18a	D;E	Give the source of funding and the role of the funders for the present study	21
Conflicts of interest	18b	D;E	Declare any conflicts of interest and financial disclosures for all authors	21
Protocol	18c	D;E	Indicate where the study protocol can be accessed or state that a protocol was not prepared	N/A
Registration	18d	D;E	Provide registration information for the study, including register name and registration number, or state that the study was not registered	N/A
Data sharing	18e	D;E	Provide details of the availability of the study data	20
Code sharing	18f	D;E	Provide details of the availability of the analytical code [4]	20
**PATIENT & PUBLIC INVOLVEMENT**				
Patient & Public Involvement	19	D;E	Provide details of any patient and public involvement during the design, conduct, reporting, interpretation, or dissemination of the study or state no involvement.	N/A
**RESULTS**				
Participants	20a	D;E	Describe the flow of participants through the study, including the number of participants with and without the outcome and, if applicable, a summary of the follow -up time. A diagram may be helpful.	8, Figure 3
	20b	D;E	Report the characteristics overall and, where applicable, for each data source or setting, including the key dates, key predictors (including demographics), treatments received, sample size, number of outcome events, follow-up time, and amount of missing data. A table may be helpful. Report any differences across key demographic groups.	Suppl.Tab.1
	20c	E	For model evaluation, show a comparison with the development data of the distribution of important predictors (demographics, predictors, and outcome).	Figure 2
Model development	21	D;E	Specify the number of participants and outcome events in each analysis (e.g., for model development, hyperparameter tuning, model evaluation)	8
Model specification	22	D	Provide details of the full prediction model (e.g., formula, code, object, application programming interface) to allow predictions in new individuals and to enable third-party evaluation and implementation, including any restrictions to access or re -use (e.g., freely available, proprietary) [5]	13-17, 20
Model performance	23a	D;E	Report model performance estimates with confidence intervals, including for any key subgroups (e.g., sociodemographic). Consider plots to aid presentation.	3-5, Figure 1
	23b	D;E	If examined, report results of any heterogeneity in model performance across clusters. See TRIPOD Cluster for additional details [3].	N/A
Model updating	24	E	Report the results from any model updating, including the updated model and subsequent performance	N/A
**DISCUSSION**				
Interpretation	25	D;E	Give an overall interpretation of the main results, including issues of fairness in the context of the objectives and previous studies	5-8
Limitations	26	D;E	Discuss any limitations of the study (such as a non -representative sample, sample size, overfitting, missing data) and their effects on any biases, statistical uncertainty, and generalizability	7-8
Usability of the model in the context of current care	27a	D	Describe how poor quality or unavailable input data (e.g., predictor values) should be assessed and handled when implementing the prediction model	N/A
	27b	D	Specify whether users will be required to interact in the handling of the input data or use of the model, and what level of expertise is required of users	Figure 4
	27c	D;E	Discuss any next steps for future research, with a specific view to applicability and generalizability of the model	8

From: Collins GS, Moons KGM, Dhiman P, et al. *BMJ* 2024;385:e078378. doi:10.1136/bmj-2023-078378

[4] This relates to the analysis code, for example, any data cleaning, feature engineering, model building, evaluation.
[5] This relates to the code to implement the model to get estimates of risk for a new individual.
